# AI-designed OpenCRISPR-1 enables efficient targeted mutagenesis and prime editing in rice

**DOI:** 10.1016/j.abiote.2026.100054

**Published:** 2026-05-20

**Authors:** Ajay Gupta, Rabia Ahuja, Bo Liu, Mark Adero, Dung Thi Pham, Wolf B. Frommer, Bing Yang

**Affiliations:** a Division of Plant Science and Technology, Bond Life Sciences Center, University of Missouri, Columbia, MO, 65211, USA; b Department of Molecular Biology and Applied Biotech, Vietnam National University of Agriculture, Hanoi, 12406, Vietnam; c Heinrich Heine University Düsseldorf, Faculty of Mathematics and Natural Sciences, Institute for Molecular Physiology, Düsseldorf, 40225, Germany; d Institute for Transformative Bio-Molecules (WPI-ITbM), Nagoya University, Nagoya, 464-8601, Japan; e Donald Danforth Plant Science Center, St. Louis, MO, 63132, USA

**Keywords:** CRISPR-Cas9, AI designed OpenCRISPR-1, Freedom to operate (FTO), Bacterial blight, Rice (*Oryza sativa*), Prime editing, Open source

## Abstract

Recent advances in generative artificial intelligence (AI) have enabled the *de novo* design of genome-editing nucleases. For example, OpenCRISPR-1 offers an open-source alternative to naturally evolved CRISPR systems and expands the "freedom to operate" (FTO). Here, we report the development and systematic validation of a monocot-optimized OpenCRISPR-1-based genome-editing ecosystem in rice (*Oryza sativa*). By targeting the *OsSWEET* susceptibility (*S*) gene family, we demonstrate that OpenCRISPR-1 supports robust multiplexed editing in both rice calli and stable T0 plants, with mutation frequencies reaching 100% in some samples. Deep sequencing revealed that the OpenCRISPR-1 mutational landscape mirrors that of *Streptococcus pyogenes* Cas9 (SpCas9), facilitating the development of predictable loss-of-function alleles that confer broad-spectrum resistance to bacterial blight. To develop a fully open-source platform, we integrated an AI-designed Open sgRNA scaffold (OpsgRNA), which maintained high editing efficacy across multiple target loci, into our editing system. Furthermore, we expanded the toolkit by engineering OpenPE6c, an OpenCRISPR-1-based prime editing system. OpenPE6c exhibited precise editing rates in rice protoplasts comparable to that of canonical SpCas9-PE6c while significantly reducing imprecise byproducts, suggesting that the AI-designed nuclease has enhanced fidelity. Our results establish OpenCRISPR-1 as a versatile, high-performance, public-access platform for advanced plant genome engineering, offering a transparent framework for the global democratization of precision crop breeding.

## 1 Introduction

To date, CRISPR-Cas9 has been applied to more than 60 plant species, targeting over 110 commercial varieties and hundreds of agronomically important genes [1]. Despite the widespread use of this system, only a few genome-edited crop products have reached commercialization [2]. This gap highlights key barriers limiting the deployment of CRISPR-Cas9 for crop improvement. Major challenges include the need to optimize prokaryotic nucleases such as CRISPR-Cas9 for plant systems, constraints imposed by intellectual property associated with the original CRISPR-Cas9 platform, and evolving regulatory frameworks governing genome-edited crops [[3], [4], [5]]. While regulatory policies continue to develop, recent advances in generative artificial intelligence (AI) have begun to address the first two challenges [6].

Breakthroughs in protein language models (LMs) now enable the *de novo* design of CRISPR-like nucleases. OpenCRISPR-1, an AI-generated, chemically synthesized nuclease, represents a significant departure from evolutionarily derived Cas9 proteins such as *Streptococcus pyogenes* Cas9 (SpCas9) [6]. By exploring protein sequence space unconstrained by natural selection, AI-designed enzymes offer the potential for enhanced specificity and expanded freedom to operate (FTO). Using LM-guided design, Ruffolo et al. generated hundreds of candidate Cas nucleases and experimentally evaluated their activity. Among the 209 Cas nucleases tested, one exhibited robust genome-editing performance, leading to the development of OpenCRISPR-1, which incorporates an engineered OpenCas9 (OpCas9) scaffold [6].

Although OpenCRISPR-1 shows promise, its performance in plant systems remains largely unexplored. In particular, the indel (insertion/deletion) profiles generated by OpCas9 relative to SpCas9 in plants have not been characterized. Furthermore, the compatibility of OpenCRISPR-1 with Prime Editing (PE), a versatile genome-editing approach for creating precise nucleotide substitutions, insertions, and deletions without double-strand breaks (DSBs), has not been assessed. PE relies on a Cas9 nickase fused to a reverse transcriptase and programmed by PE guide RNAs (pegRNAs) to install defined sequence changes [7]. Thus, the optimization of OpCas9 in terms of both nuclease activity and PE applications is critical for the development of open-source genome engineering platforms in plants.

Rice (*Oryza sativa*), a staple food crop cultivated for one-fifth of the global population, serves as a prime model for genome engineering in cereals [8]. SpCas9 exhibits high activity in rice for both indel generation and prime editing, making it an ideal system to benchmark new nucleases. In addition, rice offers several experimental advantages, including temperature-responsive editing efficiency, well-characterized agronomic targets, and strong translational relevance to other monocot crops [9].

In this study, we developed a monocot-optimized OpenCRISPR-1 system based on OpCas9 and systematically evaluated its genome-editing performance in rice. To evaluate the OpenCRISPR-1 system, we used targets from the *SUGARS WILL EVENTUALLY BE EXPORTED TRANSPORTER* (*OsSWEET*) gene family, which are well-characterized genes involved in susceptibility to bacterial blight caused by *Xanthomonas oryzae* pathovar *oryzae* (*Xoo*) [10]. The OpenCRISPR-1 system displayed editing efficiency and indel landscape comparable to those of SpCas9 in both rice callus lines and regenerated plants. The edits generated by OpenCRISPR-1 were stably inherited and conferred robust broad-spectrum resistance against *Xoo*. In addition, we engineered an alternative guide RNA scaffold, termed Open guide RNA (OpsgRNA), which shares approximately 80% sequence similarity with the canonical sgRNA scaffold. OpensgRNA performed comparably to the standard sgRNA. Finally, we developed the OpenPE6c system by fusing OpCas9 to the evoTf1 reverse transcriptase. In rice protoplasts, OpenPE6c achieved precise editing efficiencies comparable to those of the canonical PE6c system while exhibiting lower frequencies of unintended editing byproducts.

## 2 Results

### 2.1 Development of the AI-designed OpenCRISPR-1 platform for monocot genome engineering

**Fig. 1:**
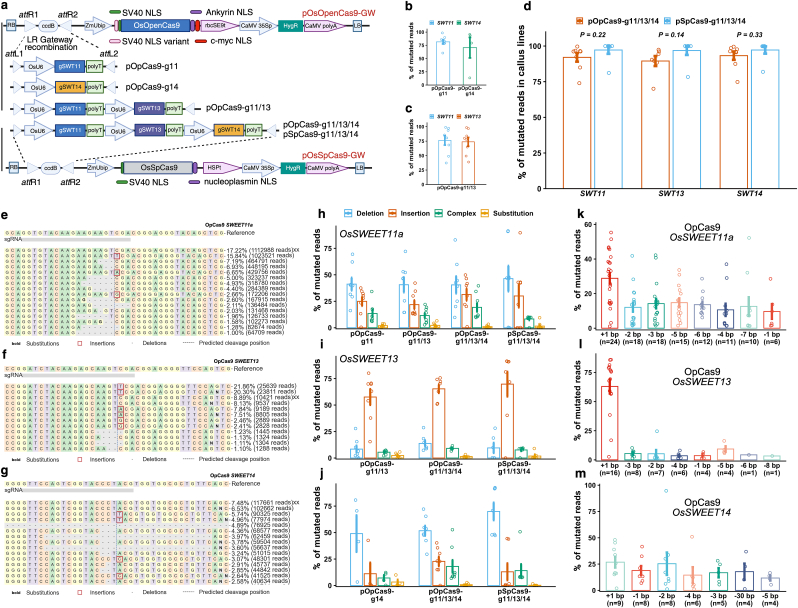
Evaluation of the editing efficiencies and mutational profiles of OpenCRISPR-1 in rice calli**.a** Schematic representation of the binary vector system. The maps at the top and bottom show the destination vectors pOsOpenCas9-GW and pOsSpCas9-GW, respectively, featuring specific NLS configurations and regulatory elements (*ZmUbi1* promoter, rbcS E9/HSP terminators). The middle panel illustrates Gateway LR recombination of sgRNA entry clones into single, dual, and triple-plex expression configurations. **b–d** Comparison of mutation frequencies in rice calli for single-plex **(b)**, duplex **(c)**, and triplex **(d)** constructs. Each data point represents an individual callus sample. Bars indicate the mean percentage of mutated reads, and error bars represent the standard error of the mean (SEM). *P*-values in **(d)** indicate no significant difference between OpCas9 and SpCas9 across the three target sites (Tukey's HSD). **e–g** CRISPResso2 visualization of the mutational landscape for *OsSWEET11a***(e)**, *OsSWEET13***(f)**, and *OsSWEET14***(g)**. The wild-type (WT) reference sequence is shown at the top, with the sgRNA indicated by a gray bar. Substitutions are shown in bold, insertions are enclosed in red boxes, and deletions are represented by dashes. The vertical dashed line indicates the predicted cleavage position. Relative read percentages and counts are provided on the right; "xx" denotes the WT allele. **h–j** Categorization of editing outcomes into deletions, insertions, complex edits, and substitutions for *OsSWEET11a***(h)**, *OsSWEET13***(i)**, and *OsSWEET14***(j)** across different construct configurations. **k–m** Frequency distribution of specific indel sizes (e.g., +1 bp, −3 bp, −30 bp) at each target locus. The number of unique alleles (*n*) contributing to each category is indicated below the *x*-axis. Data points represent the frequency of individual alleles across callus lines. Error bars represent SEM.

To evaluate the efficacy of the AI-designed OpenCRISPR-1 system in plants, we engineered a monocot-optimized version, pOsOpenCas9-GW. This construct was derived from the previously established pSpCas9-GW platform, in which *SpCas9* was replaced with a codon-optimized *OpenCas9* (*OpCas9*) driven by the maize *ubiquitin1* promoter (*ZmUbi1*). To ensure robust nuclear import, *OpCas9* was flanked by four nuclear localization signals (NLSs), with two each at the N- and C-termini. The final binary vector utilized a Gateway-compatible *ccdB* gene cassette flanked by *att*R recombination sites, enabling efficient integration of sgRNA modules (Fig. 1a).

To assess the editing activity of OpenCRISPR-1, we developed single-plex and multiplex binary vectors targeting members of the rice *OsSWEET* gene family (Fig. 1a). Unlike previous studies targeting promoter regions [11], we designed sgRNAs to target the coding regions of *OsSWEET11a*, *OsSWEET13*, and *OsSWEET14* to induce loss-of-function indel mutations (Fig. S1). Each sgRNA was expressed under the control of the *OsU6* promoter (Fig. 1a). Using Gateway LR recombination, we generated constructs in single (g11, g14), dual (g11/13), and triple (g11/13/14) sgRNA configurations (Fig. 1a). A standard SpCas9 triple-target construct (g11/13/14) served as the benchmark for comparative analyses of editing efficiency and mutational profiles (Fig. 1a).

We individually delivered the five constructs into immature embryos of rice cultivar Kitaake via *Agrobacterium*-mediated transformation. After two rounds of hygromycin selection, we harvested representative callus lines for molecular analysis. We amplified the target loci by PCR and analyzed them using Illumina next-generation sequencing (NGS). Because transformed callus lines might consist of heterogeneous cell populations, we quantified editing efficiency as the proportion of mutated reads among total reads. OpenCRISPR-1 achieved robust editing across all targets, with mutation rates consistently exceeding 60% and reaching near-saturation (100%) in some samples (Fig. 1b–d). Notably, dual- and triple-targeting constructs exhibited synchronous co-editing of multiple loci at comparable frequencies (Fig. 1c and d). OpCas9 and SpCas9 displayed nearly identical editing efficiencies across *OsSWEET11a*, *OsSWEET13*, and *OsSWEET14*, demonstrating that OpenCRISPR-1 is a highly viable alternative to canonical SpCas9 for plant genome engineering (Fig. 1d).

To further characterize the mutational spectrum, we pooled NGS reads based on target genes for each nuclease. Similar to SpCas9, OpCas9-induced mutations were centered at the 3rd and 4th nucleotides upstream of the Protospacer Adjacent Motif (PAM), indicating that the AI-designed nuclease generates DSBs at the canonical SpCas9 cleavage position (Fig. 1e–g and Fig. S2). The relative frequencies of deletions, insertions, substitutions, and complex edits were remarkably similar between OpCas9 and SpCas9 (Fig. 1h–j). While editing outcomes were locus-dependent, with deletions predominating in *OsSWEET11a* and *OsSWEET14* and insertions more frequent in *OsSWEET13*, the behavior of the two enzymes remained highly consistent (Fig. 1h–j). Detailed sequence analysis revealed 1-bp insertions as the most common editing outcome (particularly at *OsSWEET13*), followed by small deletions ranging from 1 to 8 bp. Deletions larger than 8 bp were rare and observed only at *OsSWEET14* (Fig. 1k-m), while insertions larger than 1 bp were not detected. Among 1-bp insertions, thymine was incorporated most frequently, followed by adenine, guanine, and cytosine (Fig. 1e–g). Collectively, these data demonstrate that OpCas9 recapitulates the activity and mutational characteristics of SpCas9, offering a robust, open-source platform for DSB-mediated site-specific mutagenesis in crops.

### 2.2 OpenCRISPR-1 facilitates high-frequency single and multiplexed editing in stable rice transformants

We next evaluated the editing outcomes in T0 plants derived from five independent transformation experiments. To detect targeted mutations, we amplified genomic regions flanking the sgRNA-target sites in *OsSWEET11a* and *OsSWEET1*3 by PCR and examined them using restriction fragment length polymorphism (RFLP) analysis, leveraging the disruption of target-associated restriction sites. For *OsSWEET14*, all samples underwent deep-amplicon sequencing.

**Fig. 2:**
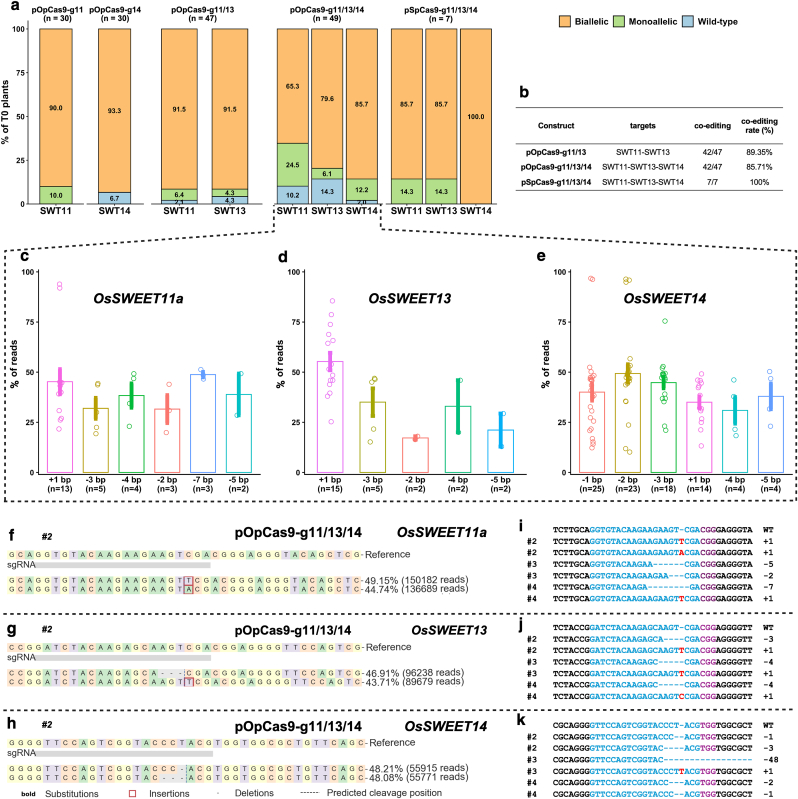
OpenCRISPR-1 facilitates high-efficiency single-plex and multiplex editing in T0 rice plants. **a** Distribution of mutation zygosity in T0 lines across five independent transformation experiments. The percentages of biallelic (orange), monoallelic (green), and wild-type (blue) plants are indicated within the stacked bars. Construct names and total T0 lines (*n*) are provided above each bar. Target genes are abbreviated as *SWT11* (*OsSWEET11a*), *SWT13* (*OsSWEET13*), and *SWT14* (*OsSWEET14*). **b** Summary of co-editing efficiencies for duplex and triplex targeting experiments. The table lists the construct, targeted loci, the ratio of co-edited plants to total lines, and the calculated co-editing frequency. **c–e** Mutational spectra in T0 plants harboring the triplex pOpCas9-g11/13/14 construct for *OsSWEET11a***(c)**, *OsSWEET13***(d)**, and *OsSWEET14***(e)**. Bars represent the mean percentage of each mutation type, and individual data points indicate the frequency of specific mutated alleles. Error bars represent the standard error of the mean (SEM). **f–h** Representative CRISPResso2 analysis of deep-amplicon sequencing data for line #2. Reference sequences are shown at the top, with sgRNA targets indicated by gray bars. Substitutions (bold), insertions (red boxes), and deletions (dashes) are highlighted. Relative read percentages and total counts are provided on the right. **i–k** Representative T0 genotypes for lines #2, #3, and #4 across the three target loci. The sgRNA sequence is highlighted in blue and the PAM in purple. Red letters indicate insertions, while dashes represent deletions. The specific allelic outcomes (e.g., +1, −5) for each sequence are shown on the right.

In single-plex editing experiments using OpenCRISPR-1, mutation rates reached 100% for *OsSWEET11a* and 93.3% for *OsSWEET14* (Fig. 2a, Tables S1 and S2). In duplex editing of *OsSWEET11a* and *OsSWEET13*, mutation rates were 97.9% and 95.7%, respectively, with a co-editing frequency of 89% (Fig. 2a and b, Table S3). For triplex editing of *OsSWEET11a, OsSWEET13*, and *OsSWEET14*, OpenCRISPR-1 maintained high editing efficiency, with mutation rates of 89.8%, 85.7%, and 98%, respectively, and a triple-gene co-editing rate of 85% (Fig. 2a and b, Table S4). While SpCas9 achieved a 100% mutation rate across all targets (Fig. 2a and b, Table S5), OpenCRISPR-1 exhibited similarly robust activity, with the majority of edited plants harboring biallelic mutations.

To further characterize the mutation spectrum in T0 plants, we performed deep-amplicon sequencing on several triplex-edited lines. Consistent with our observations in rice calli, 1-bp insertions and small deletions were the predominant outcomes generated by both OpenCRISPR-1 and SpCas9. Specifically, *OsSWEET11a* and *OsSWEET13* were enriched for 1 bp insertions (+1-bp), followed by −3-bp and other small deletions. By contrast, mutations in *OsSWEET14* were more evenly distributed among −1-bp, −2-bp, −3-bp, and +1-bp events (Fig. 2c–e and Table S1–S5).

Importantly, most T0 plants carried frameshift mutations (indels not divisible by three) capable of disrupting gene function (Fig. 2f–k and Table S1–S5). The mutational spectra of OpenCRISPR-1 and SpCas9 were nearly identical, confirming that both nucleases induce similar types of DSBs (Fig. S2 and S3, and Table S1–S5). Collectively, these results demonstrate that OpenCRISPR-1 is highly active in rice and is capable of inducing biallelic and multiplex mutations at rates comparable to SpCas9, enabling the rapid generation of complete triple-knockout lines.

### 2.3 OpenCRISPR-1-edited rice lines exhibit broad-spectrum resistance to bacterial blight

To evaluate the functional consequences of OpenCRISPR-1-induced mutations, we challenged the biallelically edited T0 lines with various *Xanthomonas oryzae* pv. *Oryzae* (*Xoo*) strains. The rice *OsSWEET11a, OsSWEET13*, and *OsSWEET14* genes are known targets of specific *Xoo* Transcription Activator-Like (TAL) effectors: PthXo1, PthXo2, and both AvrXa7 and PthXo3, respectively [11]. In this pathosystem, resistance is typically achieved by disrupting the Effector Binding Elements (EBEs) in the promoters or coding regions of these susceptibility (*S*) genes, thereby preventing the pathogen from hijacking host sugar transport pathways [10].

**Fig. 3:**
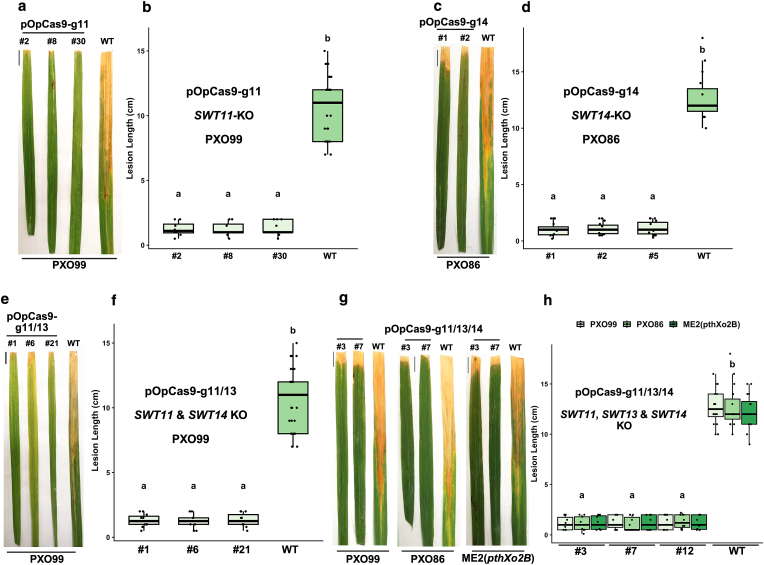
OpenCRISPR-1-mediated *OsSWEET* knockouts confer robust resistance to bacterial blight in T0 rice plants**.a–b** Representative phenotypes **(a)** and lesion lengths **(b)** of T0 lines harboring biallelic *OsSWEET11a* knockouts (pOpCas9-g11) compared to wild-type (WT) Kitaake following inoculation with *Xoo* strain PXO99. **c–d** Phenotypic responses **(c)** and lesion lengths **(d)** of *OsSWEET14* knockout lines (pOpCas9-g14) and WT controls inoculated with strain PXO86. **e–f** Validation of the resistance of duplex *OsSWEET11a/OsSWEET13* knockout lines (pOpCas9-g11/13) and WT inoculated with strain PXO99. **g–h** Broad-spectrum resistance assay for triplex *OsSWEET11a/OsSWEET13/OsSWEET14* knockout lines (pOpCas9-g11/13/14) and WT plants inoculated with three *Xoo* strains: PXO99, PXO86, and ME2 (*pthXo2B*).

We inoculated the T0 lines with three distinct *Xoo* strains, each carrying a cognate effector for a specific *OsSWEET* gene. Plants harboring mutations in *OsSWEET11a* exhibited robust resistance against strain PXO99, which depends on its *pthXo1* gene for virulence (Fig. 3a and b). Likewise, *OsSWEET14* mutants were resistant to strain PXO86 carrying *avrXa7* (Fig. 3c and d). Double knockout lines of *OsSWEET11a* and *OsSWEET14* challenged with PXO99 exhibited a resistant phenotype (Fig. 3e and f). Most notably, the triple-knockout lines displayed broad-spectrum resistance against all three strains tested, which is consistent with phenotypes previously reported for multiplex *SWEET* knockout lines (Fig. 3g and h) [12]. Collectively, these results demonstrate that the indel mutations generated by the OpenCRISPR-1 system successfully produced loss-of-function alleles that effectively blocked TAL effector-mediated susceptibility, thereby providing effective, broad-spectrum resistance against multiple strains of *Xoo*.

In all boxplots, horizontal lines represent the median, and individual data points indicate measurements from *n* = 3–5 leaves per plant. Lesion lengths were recorded 12 days post-inoculation. Scale bars = 1 cm. Different lowercase letters (a, b) denote statistically significant differences (*P* < 0.05), as determined by Tukey's HSD test.

### 2.4 Edits generated by OpenCRISPR-1 are stably inherited

To evaluate the heritability of OpenCRISPR-1-induced mutations, we advanced several edited T0 lines to the T1 generation. All of these T0 lines successfully transmitted the edited alleles to their progeny in accordance with the expected Mendelian inheritance patterns (Tabel 1). T0 lines identified as biallelic mutants gave rise to fixed homozygous mutants in the T1 progeny. Notably, a T0 line with a monoallelic mutation in *OsSWEET13* (pOpCas9-g11/13/14 #11) segregated in the T1 generation into homozygous, heterozygous, and wild-type progeny, which is consistent with Mendelian segregation (Table 1). These results demonstrate that OpenCRISPR-1-mediated edits are stable and successfully integrated into the germline.

**Table 1:** Inheritance of edited alleles in the T1 progeny of selected edited T0 lines.

T0 line	T0 genotype	T1 genotype									Transgene free
		OsSWEET11a			OsSWEET13			OsSWEET14			
	SWT11/SWT13/SWT14	Ho	He	WT	Ho	He	WT	Ho	He	WT	
pOpCas9-g11 #2	Bi/WT/WT	16	0	0	-	-	-	-	-	-	3
pOpCas9-g11 #8	Bi/WT/WT	16	0	0	-	-	-	-	-	-	3
pOpCas9-g11 #22	Bi/WT/WT	6	0	0	-	-	-	-	-	-	3
pOpCas9-g11 #35	Bi/WT/WT	15	0	0	-	-	-	-	-	-	15

pOpCas9-g14 #6	WT/WT/Bi	-	-	-	-	-	-	6	0	0	2
pOpCas9-g14 #10	WT/WT/Bi	-	-	-	-	-	-	16	0	0	4
pOpCas9-g14 #12	WT/WT/Bi	-	-	-	-	-	-	16	0	0	6

pOpCas9-g11/13 #3	Bi/Bi/WT	3	0	0	3	0	0	-	-	-	0
pOpCas9-g11/13 #6	Bi/Bi/WT	13	0	0	13	0	0	-	-	-	6
pOpCas9-g11/13 #46	WT/WT/WT	0	0	11	0	0	11	-	-	-	5
pOpCas9-g11/13 #52	Bi/Bi/WT	7	0	0	7	0	0	-	-	-	6

pOpCas9-g11/13/14 #11	Bi/Mo/Bi	8	0	0	2	5	1	8	0	0	2
pOpCas9-g11/13/14 #30	WT/WT/Bi	0	0	16	0	0	16	16	0	0	6

Bi: Biallelic, Mo: Monoallelic, WT: Wild type, Ho: Homozygous edit, He: Heterozygous edit, -: Not tested.

We also monitored a specific T0 line that carried the OpenCRISPR-1 transgene but showed no detectable editing at the target locus. Screening of its T1 progeny revealed no newly generated mutations, suggesting that the absence of editing was likely caused by transgene silencing. To identify transgene-free lines, we performed PCR-based genotyping to detect T-DNA. In all lines examined except one, we successfully recovered transgene-free segregants that retained the desired knockout mutations (Table 1). These results highlight the robustness of the OpenCRISPR-1 system for generating stable, heritable genome edits and demonstrate its utility for the rapid production of non-transgenic, genome-edited rice lines within a single generation following transformation.

### 2.5 Modular integration of open-source sgRNA scaffolds produces a fully AI-designed CRISPR-Cas9 ecosystem in monocots

**Fig. 4:**
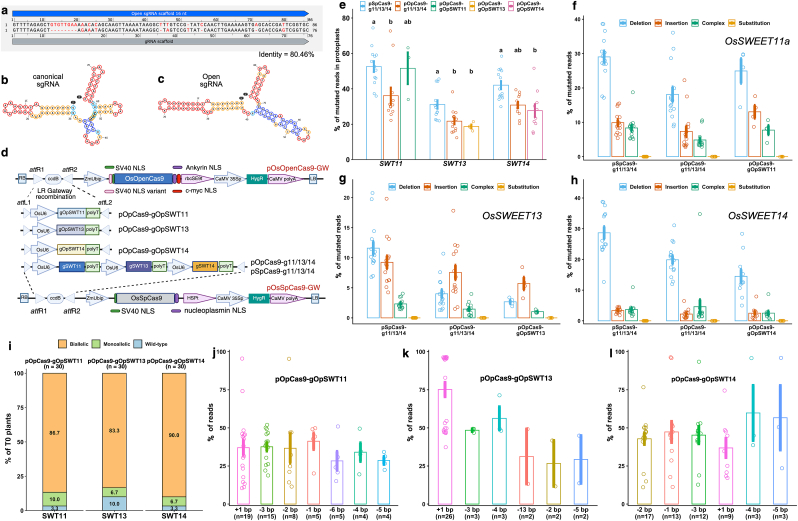
The AI-designed open sgRNA scaffold (OpsgRNA) maintains high editing efficacy in rice. **a** Sequence alignment between the canonical sgRNA scaffold and the open sgRNA scaffold. Red letters indicate nucleotide polymorphisms; the overall sequence identity is 80.46%. **b–c** Predicted secondary structures of the canonical **(b)** and open **(c)** sgRNA scaffolds generated via the ViennaRNA algorithm. The color gradient represents base-pairing probability (red: >90%; blue: <50%). **d** Diagram of the binary vector configurations used for protoplast and T0 assays. The OpsgRNA modules were integrated into OpCas9 destination vectors via Gateway LR recombination. **e** Editing frequencies in rice protoplasts across three *OsSWEET* targets for the five indicated nuclease/scaffold configurations. Each data point represents an independent protoplast transfection. Bars indicate the mean percentage of mutated reads; error bars represent SEM. Different lowercase letters denote statistically significant differences (*P* < 0.05, Tukey's HSD). **f–h** Distribution of mutation types (deletions, insertions, complex, and substitutions) in rice protoplasts for *OsSWEET11a***(f)**, *OsSWEET13***(g)**, and *OsSWEET14***(h)**. **i** Zygosity distribution in T0 lines generated using the OpCas9-OpsgRNA system. Stacked bars indicate the percentages of biallelic (orange), monoallelic (green), and wild-type (blue) plants. The number of T0 lines (*n*) is provided above each bar. **j–l** Mutational spectra in T0 lines for *OsSWEET11a***(j)**, *OsSWEET13***(k)**, and *OsSWEET14***(l)**. Bars represent the mean percentage of each indel size; individual data points represent unique mutated alleles. Error bars represent SEM.

In addition to the AI-designed OpCas9 nuclease, Ruffolo et al. designed two open-source sgRNA scaffolds that share approximately 80% sequence similarity while maintaining conserved RNA secondary structures and activity comparable to the canonical SpCas9 sgRNA scaffold (Fig. 4a–c). To evaluate the compatibility of these AI-designed components in plants, we integrated one of the open-source scaffolds (OpsgRNA) into our editing system by replacing the canonical sgRNA scaffold and targeting the three *OsSWEET* genes individually in both rice protoplasts and stable transgenic plants (Fig. 4d).

In rice protoplast assays, we compared OpCas9 constructs carrying either the OpsgRNA or the canonical scaffold, with SpCas9 and its canonical sgRNA serving as the control. Consistent with observations from T0 lines, OpCas9 exhibited slightly lower editing efficiencies than SpCas9. Importantly, replacement of the canonical scaffold with OpsgRNA did not further reduce editing activity, indicating that both scaffold architectures perform equivalently in rice cells (Fig. 4e).

The mutational profiles generated by the three editor configurations were largely similar for *OsSWEET11a* and *OsSWEET14* (Fig. 4f–h and Fig. S4). However, at *OsSWEET13*, SpCas9 preferentially generated deletion events, whereas both OpCas9 configurations (canonical and open scaffold) predominantly induced insertions (Fig. 4g and Fig. S4). Overall, these results demonstrate that the canonical gRNA scaffold can be replaced with an open-source alternative without compromising editing efficacy or altering overall editing outcomes.

To further validate the system in stable transformants, we generated T0 rice plants using the OpCas9-OpsgRNA configuration. High mutation rates were achieved at all target loci, reaching 96.7%, 90%, and 96.7% for *OsSWEET11a, OsSWEET13*, and *OsSWEET14*, respectively (Fig. 4i). These efficiencies were highly comparable to those obtained with the canonical scaffold. The mutational profiles were also consistent with previous observations: edits at *OsSWEET11a* and *OsSWEET13* were dominated by +1-bp and −3-bp edits, while *OsSWEET14* primarily accumulated –1-bp, −2-bp, and −3-bp indels (Fig. 4j-l, and Table S6–S8). As observed in earlier experiments, most of these lines carried frameshift mutations predicted to generate knockouts, except for the in-frame −3-bp deletions. Collectively, these data establish that the fully AI-designed OpenCRISPR system, consisting of both an engineered nuclease and an open-source scaffold, functions robustly in rice, providing a practical platform for genome engineering in monocot crops.

### 2.6 Expansion of the OpenCRISPR-1 toolkit for precision genome editing via a high-fidelity OpenPE6c system

**Fig. 5:**
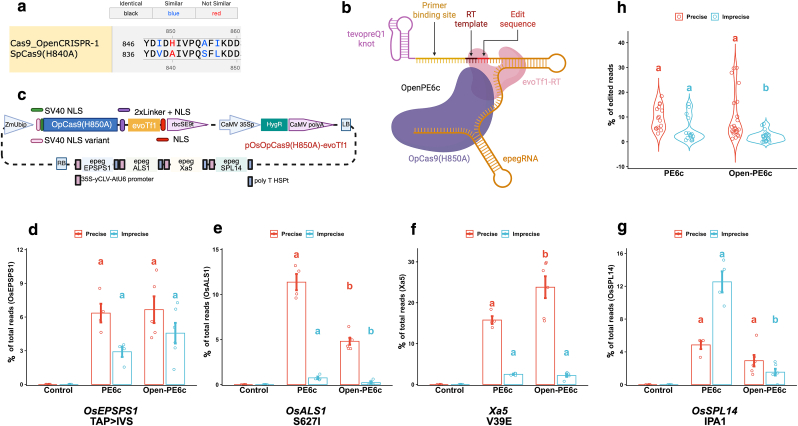
High-precision prime editing in rice protoplasts using the OpenPE6c system. **a** Amino acid sequence alignment of the HNH nuclease domains of OpCas9 and SpCas9. Identical residues are shown in black, similar residues in blue, and non-conserved residues in red. The H850A mutation in OpCas9 (corresponding to SpCas9 H840A) is indicated to denote the creation of the Cas9 nickase. **b** Schematic representation of the OpenPE6c complex. The OpCas9(H850A) nickase is fused to the evolved Tf1 reverse transcriptase (evoTf1-RT). The engineered pegRNA (epegRNA) features a tevopreQ1 3′ structural motif, a primer binding site (PBS), and an RT template for installing the desired edit. **c** Diagram of the PE binary vector and multiplex epegRNA expression cassette. Individual epegRNAs targeting *OsEPSPS1*, *OsALS1*, *Xa5*, and *OsSPL14* are driven by the 35S-yCLV-AtU6 promoter system. **d–g** Comparative analysis of precise (red) and imprecise (blue) editing frequencies in rice protoplasts across four target loci. Precise edits represent the intended template-directed changes, while imprecise edits represent unintended indels or byproducts. Each data point represents an independent transfection (*n* ≥ 4); bars indicate the mean percentage of total reads, and error bars represent SEM. Significant differences (*P* < 0.05, Tukey's HSD) between PE6c and OpenPE6c are denoted by different lowercase letters. **h** Violin plot illustrating the distribution of combined precise and imprecise editing frequencies across all four targets. The width of the violin represents the kernel density of the data points. Statistically significant reductions in imprecise editing by OpenPE6c compared to PE6c are indicated by lowercase letters.

To determine whether OpCas9 could be adapted for precision genome editing, we evaluated its performance within a PE framework. PE typically utilizes a Cas9 nickase (nCas9) fused to a reverse transcriptase (RT) to install template-directed sequence modifications without generating DSBs [7]. In SpCas9, the H840A mutation in the HNH nuclease domain abolishes cleavage of the target DNA strand, thereby converting the nuclease into a nickase. By aligning the amino acid sequences of SpCas9 and OpCas9, we identified the corresponding HNH catalytic residue in the AI-designed nuclease and introduced the H850A substitution to generate an OpCas9 nickase (Fig. 5a, and Fig. S5).

To construct a prime editor, we fused the OpCas9(H850A) nickase to an evolved Tf1 reverse transcriptase (evoTf1) using the PE6c architecture [13], resulting in the OpenPE6c system (Fig. 5b and c). The PE6c framework was selected due to its reported 2–5-fold higher efficiency over the PE2 architecture. In addition, the compact size of evoTf1 is advantageous for maintaining efficient *Agrobacterium*-mediated transformation in rice. To benchmark OpenPE6c, we designed a multiplex construct carrying the OpenPE6c effectors together with four engineered pegRNAs (epegRNAs) targeting *OsEPSPS1*, *OsSPL14*, *OsALS1*, and *Xa5*. We directly compared the editing performance of this construct with a conventional SpCas9-based PE6c construct targeting the same four loci in rice protoplasts (Fig. 5c).

Deep-amplicon sequencing of protoplast DNA collected 48 h post-transfection revealed that OpenPE6c and the standard PE6c achieved comparable levels of precise editing, defined as the incorporation of the intended nucleotide change. Compared to PE6c, OpenPE6c displayed similar editing efficiencies at *OsEPSPS1* and *OsSPL14*, slightly lower activity at *OsALS1*, and improved performance at the *Xa5* locus (Fig. 5d–g and Fig. S6).

Crucially, we also quantified imprecise editing (unintended indels or byproducts). OpenPE6c exhibited similar byproduct levels at *OsEPSPS1* and *Xa5* but showed a notable reduction in imprecise edits at the *OsALS1* and *OsSPL14* loci compared to the standard PE6c (Fig. 5d–g and Fig. S6).

In summary, for all targets, OpenPE6c and PE6c show similar levels of precise editing activity, while OpenPE6c shows lower amounts of imprecise editing (Fig. 5h). These findings suggest that the OpCas9-based prime editor is not only functionally equivalent to SpCas9-based systems in terms of activity but may also offer enhanced accuracy by minimizing unwanted byproducts. Collectively, these results establish OpenCRISPR-1 as a versatile, high-fidelity scaffold for advanced plant genome engineering.

## 3 Discussion

The emergence of CRISPR-Cas9 and generative AI has redefined the boundaries of biological research. In this study, we demonstrated the successful convergence of these technologies by validating the utility of the AI-designed OpenCRISPR-1 system for genome engineering in monocots. Historically, adapting prokaryotic Cas nucleases for efficient activity in eukaryotic plant cells has required extensive iterative optimization [14,15]. By leveraging protein LMs to explore the sequence space beyond the constraints of natural selection, Ruffolo et al. developed OpCas9, a nuclease that differs from SpCas9 by 403 amino acids while retaining robust editing activity. Our work bridges AI-driven protein engineering with practical agricultural applications, providing a fully optimized, open-source toolkit for crop improvement.

Our systematic evaluation of monocot-optimized OpenCRISPR-1 for single-, dual-, and triple-target editing of *OsSWEET11a*, *OsSWEET13*, and *OsSWEET14* demonstrated that OpCas9 is a highly effective alternative to SpCas9. While OpenCRISPR-1 displayed slightly lower mutation frequencies in certain contexts, likely due to the reduced nuclease activity of OpCas9 relative to SpCas9, editing rates in stable T0 lines approached saturation, reaching 100% at some targets. Importantly, the mutational spectrum generated by OpCas9, dominated by 1-bp insertions and small deletions located 3–4 bp upstream of the PAM, closely mirrored that of SpCas9. These findings suggest that the AI-designed nuclease likely generates DSBs through a mechanism similar to that of SpCas9, enabling researchers to adopt this open-source platform without sacrificing the predictable editing outcomes established through decades of SpCas9 research.

One major obstacle limiting the commercialization of genome-edited crops is the complex intellectual property landscape surrounding CRISPR technologies [3,4]. By integrating the AI-designed open sgRNA scaffold (OpsgRNA) with OpCas9, we established a fully open-source genome-editing ecosystem. The robust editing performance observed in the T0 generation and the stable inheritance of edited alleles by the T1 generation demonstrate that the OpenCRISPR-1 system is a reliable vehicle for generating non-transgenic, elite crop varieties. Furthermore, the development of the OpenPE6c system significantly expands the OpenCRISPR-1 toolkit into precision genome engineering. Notably, OpenPE6c achieved precise editing efficiencies comparable to those of SpCas9-based prime editors while generating fewer imprecise byproducts, suggesting that AI-designed proteins may offer inherent advantages in editing fidelity that could be further exploited for precision plant breeding.

AI is rapidly becoming a cornerstone of modern agriculture, particularly in the realms of genomics and genome engineering. Our findings align with recent preliminary reports in alfalfa (*Medicago sativa*) and rice that established the baseline functionality of OpenCRISPR-1 [16,17]. However, our study provides a substantially deeper characterization of this system in rice, including the first detailed analysis of the mutational landscape of OpCas9 in stable transgenic plants, functional validation of disease resistance, inheritance of edited alleles, and successful decoupling of edits from the transgene.

The *OsSWEET* knockout lines generated in this study can serve both biological and agricultural purposes. Biologically, they provide broad-spectrum resistance against *Xoo* by disrupting the host susceptibility pathways hijacked by TAL effectors. Agriculturally and scientifically, they provide valuable germplasm for studying source-to-sink sugar transport and the trade-off between disease resistance and plant productivity. While the impaired seed set associated with *SWEET* knockout lines has been documented [[18], [19], [20], [21]], these materials represent an important resource for dissecting the balance between immunity and yield-related traits.

By making the monocot-optimized OpenCRISPR-1 plasmids and associated plant materials publicly available, we aim to democratize access to advanced genome-editing tools. This FTO framework enables researchers and breeders globally to innovate without the constraints imposed by conventional intellectual property barriers. As AI-driven protein design continues to advance, we anticipate the development of increasingly specialized nucleases with expanded PAM compatibility, enhanced precision, and broader utility for next-generation crop improvement.

## 4 Materials and methods

All oligos used in this study are listed in Supplementary Table S9.

### 4.1 Cloning and construct assembly

The pOsOpenCas9-GW binary vector was engineered by replacing the SpCas9 coding sequence in the pHUE411 backbone (Addgene #62203) with a monocot-optimized OpenCas9 (OpCas9) sequence (Supplementary Sequence S1). The SpCas9 sequence was excised by restriction digestion with *Sac*I and *Stu*I. Monocot codon optimized OpCas9 flanked by four nuclear localization signals (NLS) was synthesized as a synthetic DNA fragment. This fragment was integrated into the digested backbone using TEDA (T5 Exonuclease-Dependent Assembly) [22] cloning to generate the intermediate plasmid pAG818 (ZmUbi1OpCas9; OsU3sgRNA). To establish Gateway-compatibility, the *OsU3* cassette was removed using *Hind*III and *Pme*I digestion and replaced with an attR1-*ccdB*-attR2 destination cassette via TEDA cloning, resulting in the final vector, pAG819 (pOsOpenCas9-GW).

For guide RNA expression, the entry vector pENTR4-sgRNA carrying the *OsU6* promoter was used. Single, dual, and triple *OsSWEET* guide RNA target sequences were synthesized as oligonucleotides (IDT) and integrated into the entry vector using Golden Gate assembly. To evaluate the OpsgRNA architecture (Supplementary Sequence S2), a specialized entry vector was synthesized (Twist Bioscience) in which the canonical sgRNA scaffold was replaced with the AI-designed open scaffold resulting in pAG902. Final binary constructs were assembled by mobilizing sgRNA expression cassettes from the entry clones into the pOsOpenCas9-GW or pOsSpCas9-GW destination vectors via recombination using LR Clonase (Thermo Fisher Scientific).

The OpenPE6c prime editor (pAG875) was derived from the pRA1 (PE6c) backbone (ZmUbi1SpCas9(H840A)-evoTf1-GW, unpublished). The SpCas9(H840A) nickase sequence was excised using *Acc65*I and *Ale*I and replaced with a synthesized OpCas9(H850A) nickase via TEDA cloning. For the PE assays, four engineered pegRNA (epegRNA) expression cassettes targeting *OsEPSPS1*, *OsALS1*, *Xa5*, and *OsSPL14* were mobilized into the OpenPE6c and PE6c (control) destination vectors using Gateway LR recombination.

### 4.2 Plant materials, bacterial strains, medium, and growth conditions

All genome-editing experiments were conducted using the *japonica* rice cultivar Kitaake (*O. sativa* L. ssp. *japonica*). Rice plants were cultivated in greenhouses and controlled-environment growth chambers under a 12-h light/12-h dark photoperiod, with day/night temperatures of 30°C and 28°C, respectively. Relative humidity was maintained between 60% and 75%.

The *Xanthomonas oryzae* pv*. Oryzae* (*Xoo*) strains used for disease assays were obtained from the Yang laboratory collection. *Xoo* strains were cultured on Tryptone Sucrose Agar (TSA) medium (10 g/L tryptone, 10 g/L sucrose, 1 g/L glutamic acid, and 15 g/L Difco agar) at 28°C. *Escherichia coli* strains DB3.0 (for propagating *ccdB*-carrying plasmids) and EPI300 (for all other constructs) used for cloning, as well as *Agrobacterium tumefaciens* strain EHA105 used for plant transformation, were cultured in Luria-Bertani (LB) medium at 37°C and 28°C, respectively.

When required, media were supplemented with the appropriate antibiotics at the following concentrations for plasmid maintenance and strain selection: 25 μg/mL rifampicin, 50 μg/mL kanamycin, and 100 μg/mL spectinomycin.

### 4.3 Rice transformation and plant regeneration

Stable rice transformation was performed via *Agrobacterium*-mediated DNA delivery using embryos derived from mature Kitaake seeds following a modified protocol based on [23]. Briefly, scutellum-derived callus formation was induced on Murashige and Skoog (MS) medium supplemented with 2 mg/L 2,4-dichlorophenoxyacetic acid (2,4-D).

Embryogenic calli were co-cultivated with *Agrobacterium tumefaciens* strain EHA105 harboring the appropriate binary vectors (e.g., pOsOpenCas9-gRNA). Following co-cultivation, the calli underwent two rounds of selection (14 days each) on MS medium supplemented with 2 mg/L 2,4-D, 50 mg/L hygromycin B, and 200 mg/L Timentin to enrich stably transformed callus cells.

Hygromycin-resistant callus lines were subsequently transferred to regeneration medium (MS supplemented with 6-benzylaminopurine [BAP] and α-naphthaleneacetic acid [NAA]) to induce shoot organogenesis. Regenerated shoots were then transferred to a rooting medium (half-strength MS medium supplemented with 25 mg/L hygromycin B). After the establishment of a healthy root system, T0 plantlets were transplanted into soil and acclimated in the greenhouse under the growth conditions described above.

### 4.4 Protoplast isolation and transfection

Rice protoplasts were isolated from the leaf sheaths of 10-day-old Kitaake seedlings as previously described, with minor modifications [24]. Briefly, leaf sheaths were cut into 0.5–1.0 mm strips and vacuum-infiltrated with an enzyme solution (1.5% [w/v] cellulase RS, 0.75% [w/v] macerozyme R-10, 0.6 M mannitol, 10 mM MES [pH 5.7], 10 mM CaCl_2_, and 0.1% BSA). The digestion was carried out for 4–5 h at 28°C in the dark with gentle shaking (80 rpm). Protoplasts were released by filtration through a 40 μm nylon mesh, followed by centrifugation at 250 × *g* for 5 min. The resulting pellet was washed twice with W5 solution (154 mM NaCl, 125 mM CaCl_2_, 5 mM KCl, and 2 mM MES [pH 5.7]) and resuspended in MMG solution (0.6 M mannitol, 15 mM MgCl_2_, and 4 mM MES [pH 5.7]). Protoplast density was assessed using a hemocytometer, and viability was assessed by Evans Blue staining.

For transfection, the protoplast concentration was adjusted to 1–2×10^6^ cells/mL in MMG solution. Approximately 10 μg of high-quality plasmid DNA was mixed with 200 μL of the protoplast suspension, followed by the addition of 220 μL of freshly prepared PEG solution (40% [w/v] PEG 4000, 0.6 M mannitol, and 100 mM CaCl_2_). The mixture was incubated at room temperature for 20 min. The transfection was terminated by adding 1 mL of W5 solution, and the protoplasts were collected by centrifugation at 300 × *g* for 5 min. The cells were gently resuspended in 1 mL of WI solution (0.5 M mannitol, 20 mM KCl, and 4 mM MES [pH 5.7]) and incubated in the dark at 28°C for 48 h prior to genomic DNA extraction.

### 4.5 Genomic DNA extraction, target site amplification, and deep-amplicon sequencing

Genomic DNA was extracted from rice protoplasts, calli, T0 plants, and T1 progeny using the cetyltrimethylammonium bromide (CTAB) method. Protoplasts and calli were screened by deep-amplicon sequencing. T0 and T1 plants were screened for mutations via PCR-RFLP (Restriction Fragment Length Polymorphism) analysis and deep-amplicon sequencing. For RFLP analysis of *OsSWEET11a* and *OsSWEET13*, the *Hinc*II restriction enzyme was used, while *Bsa*AI was used for *OsSWEET14* (Fig. S1). Based on their digestion patterns, completely digested, partially digested, and undigested PCR-amplicons were classified as wild type, monoallelic/heterozygous, and biallelic/homozygous, respectively, reflecting the presence or disruption of the corresponding restriction sites within the target amplicons.

For deep-amplicon sequencing, a two-step PCR strategy was employed to generate Illumina-compatible libraries. In the first round, the 150–250 bp region surrounding each target site was amplified using gene-specific primers containing partial Illumina adapter sequences at the 5′ ends. The resulting amplicons served as templates for a second round of nested PCR using dual-barcoded Illumina indices for multiplexed sequencing. The barcoded libraries were purified using a DNA Clean & Concentrator kit (Zymo Research), quantified, and pooled in equimolar ratios. Sequencing was performed on an Illumina MiSeq platform (PE150) at the University of Missouri–Columbia Genomics Technology Core (RRID:SCR_017778). An average minimum sequencing depth of 10,000 paired-end reads per sample was obtained. For callus samples, at least four independent samples per construct were subjected to deep sequencing. For T0 lines, a minimum of five independent lines per construct were analyzed. For protoplast assays, at least four replicates were analyzed by deep sequencing.

Raw sequencing reads were demultiplexed and quality trimmed. Mutational profiles were analyzed using the CRISPResso2 pipeline [25] to quantify Non-Homologous End Joining (NHEJ) and PE outcomes. For nuclease activity, the "NHEJ" mode was used to determine the frequency and distribution of indels relative to the predicted cleavage site. For prime editing, the "PE" mode was utilized to distinguish between precise template-directed edits and imprecise byproducts. The edit rate was calculated as the percentage of modified reads (precise + imprecise) relative to the total number of aligned reads.

### 4.6 Pathogen inoculation and disease assays

Bacterial blight disease assays were performed using the leaf tip-clipping method as previously described [26]. *Xoo* strains were recovered from glycerol stocks stored at −80°C by streaking onto TSA medium and incubating at 28°C for 72 h. Bacterial cells were harvested, washed twice with sterile distilled water, and resuspended to an optical density of OD_600_ = 0.5.

For inoculation, the tips of fully expanded leaves from T0 plants were clipped using surgical scissors dipped in the bacterial suspension. Lesion lengths were measured at 12 days post inoculation (dpi). For each strain-genotype combination, three to five independent plants were used as biological replicates, with multiple leaves inoculated per plant to ensure statistical robustness.

### 4.7 Prediction of RNA structure and amino acid sequence alignment

RNA structures were predicted and amino acid sequence alignment was conducted using the built-in tools of SnapGene software.

### 4.8 Statistical analysis and data visualization

All statistical analyses and data visualizations were performed using R (v4.6.0). For comparative analysis of editing efficiencies and lesion lengths, mean values were compared using one-way analysis of variance (ANOVA). Post-hoc comparisons were conducted using Tukey's Honest Significant Difference (HSD) test (*P* < 0.05) to identify significant differences between wild-type, SpCas9-edited, and OpCas9-edited lines.

Data visualization was carried out using the ggplot2 and ggpubr packages. Sequence alignments and mutational spectra from deep sequencing data were analyzed using the rstatix package for statistical summaries and specialized bioinformatics pipelines, including CRISPResso2, as described in the corresponding Results sections.

## CRediT authorship contribution statement

**Ajay Gupta:** Writing – review & editing, Writing – original draft, Validation, Methodology, Investigation, Formal analysis, Data curation, Conceptualization. **Rabia Ahuja:** Writing – review & editing, Investigation. **Bo Liu:** Investigation. **Mark Adero:** Investigation. **Dung Thi Pham:** Investigation. **Wolf B. Frommer:** Resources. **Bing Yang:** Writing – review & editing, Supervision, Funding acquisition, Conceptualization.

## Declaration of competing interest

The authors declare that they have no known competing financial interests or personal relationships that could have appeared to influence the work reported in this paper. Author Bing Yang was not involved in the journal's review of this manuscript.

## Data Availability

The plant meterials and constructs generated in this study are available upon request.
