# Supplementary material for: AI-designed OpenCRISPR-1 enables efficient targeted mutagenesis and prime editing in rice
Source: aBIOTECH. 2026 May 20;7(3):100054. doi: 10.1016/j.abiote.2026.100054 (PMC13352068; doi:10.1016/j.abiote.2026.100054)
Supplement: Multimedia component 1 [file mmc1.pdf]

# AI-Designed OpenCRISPR-1 Enables Efficient Targeted Mutagenesis and Prime Editing in Rice

Ajay Gupta[1], Rabia Ahuja[1], Bo Liu[1], Mark Adero[1], Dung Thi Pham[1,2], Wolf B. Frommer[3,4], Bing Yang[1,5] *
[1] Division of Plant Science and Technology, Bond Life Sciences Center, University of Missouri, Columbia, MO 65211, USA
[2] Department of Molecular Biology and Applied Biotech, Vietnam National University of Agriculture, Hanoi, Vietnam
[3] Heinrich Heine University Düsseldorf, Faculty of Mathematics and Natural Sciences, Institute for Molecular Physiology, Düsseldorf, Germany
[4] Institute for Transformative Bio-Molecules (WPI-ITbM), Nagoya University, Nagoya, Japan
[5] Donald Danforth Plant Science Center, St. Louis, MO 63132, USA

* Corresponding authors
Bing Yang (yangbi@missouri.edu)

Supplementary information
Figure S1-S6
Table S1-S9
Supplementary Sequences S1-S2

## Supplementary Figures

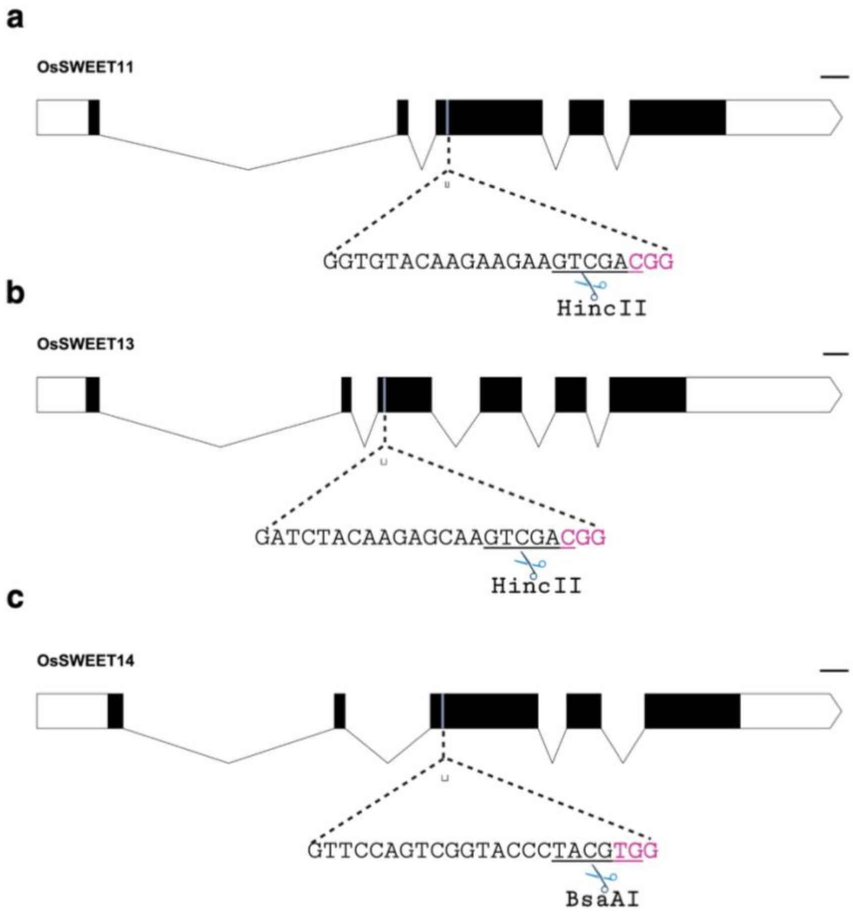


**Fig. S1: Gene models and sgRNA of *OsSWEET* genes used in this study.**
**(a-b)** Gene models of *OsSWEET11a* **(a)**, *OsSWEET13* **(b)**, and *OsSWEET14* **(c)**. Black box marks exons, V lines are introns, and white boxes are 5`-and 3`-untranslated regions (UTRs). The sgRNA region and sequence are zoomed from the third exon of three genes. PAM is marked with a purple color and restriction enzyme site around the Cas9 cutting site is underlined.

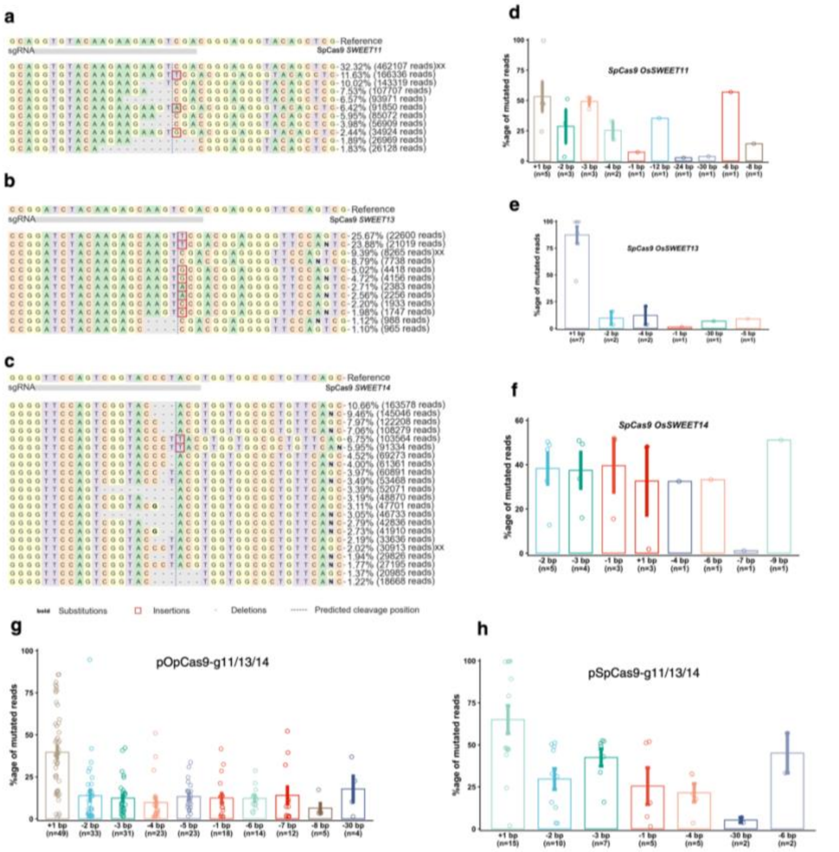


**Fig. S2: Mutational landscape of SpCas9 in rice calli.**
**(a–c)** CRISPResso2 visualization of the mutational landscape for *OsSWEET11a* **(a)**, *OsSWEET13* **(b)**, and *OsSWEET14* **(c)**. The wild-type (WT) reference sequence is shown at the top with the sgRNA indicated by a gray bar. Substitutions are shown in bold, insertions are enclosed in red boxes, and deletions are represented by dashed lines. The vertical dashed line indicates the predicted cleavage position. Relative read percentages and counts are provided on the right; "xx" denotes the WT allele.
**(d–f)** Frequency distribution of specific indel sizes (e.g., +1 bp, -3 bp, -30 bp) at each target locus. The number of unique alleles (n) contributing to each category is indicated below the x-axis. Data points represent the frequency of individual alleles across callus lines. Error bars represent SEM.
**(g-h)** Frequency distribution of specific indel sizes (e.g., +1 bp, -3 bp, -30 bp) of OpCas9 and SpCas9 for all genes combined. The number of unique alleles (n) contributing to each category is indicated below the x-axis. Data points represent the frequency of individual alleles across callus lines. Error bars represent SEM.

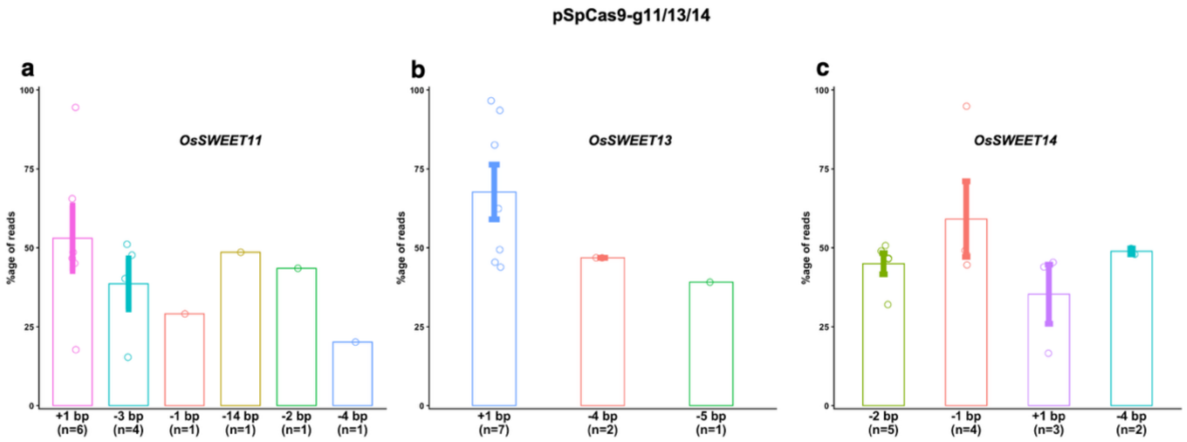


**Fig. S3: Mutational landscape of SpCas9 in rice T0 lines.**
**(a–c)** Frequency distribution of specific indel sizes (e.g., +1 bp, -3 bp, -30 bp) at each target locus. The number of unique alleles (n) contributing to each category is indicated below the x-axis. Data points represent the frequency of individual alleles across callus lines. Error bars represent SEM.

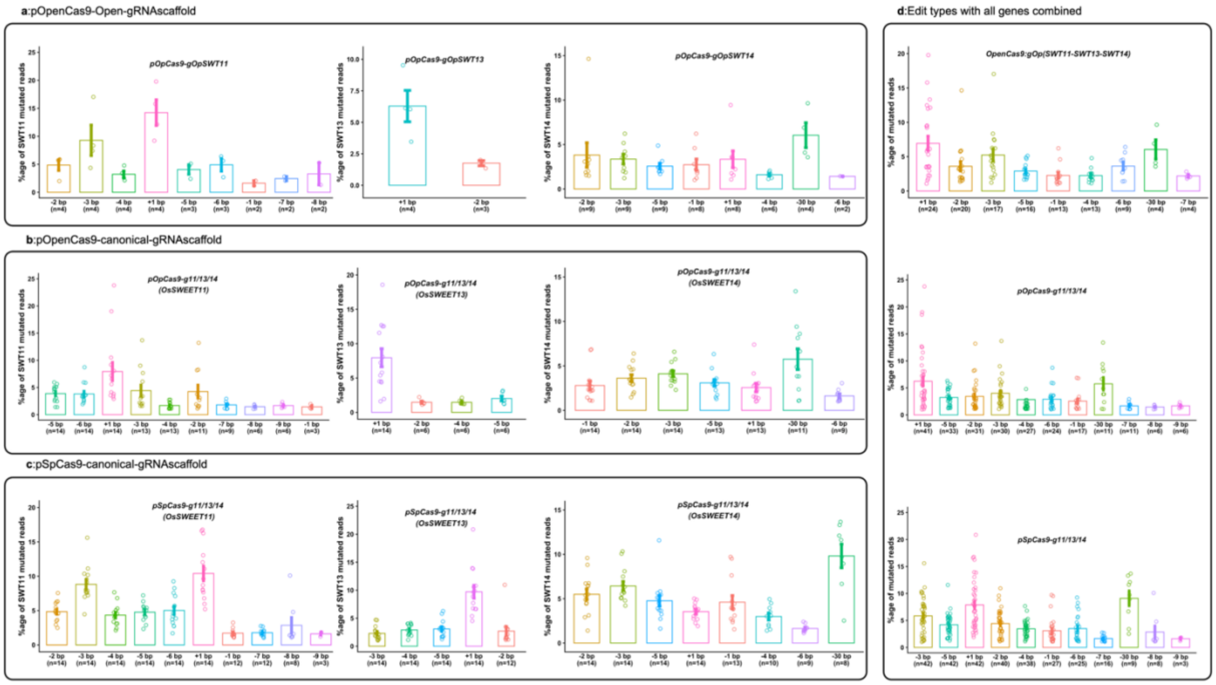


**Fig. S4: Mutational landscape of OpCas9 and SpCas9 with open sgRNA or canonical sgRNA in rice protoplasts.**

**(a–c)** Frequency distribution of specific indel sizes (e.g., +1 bp, -3 bp, -30 bp) at each target locus for OpCas9-OpsgRNA **(a)**, pOpCas9-g11/13/14 **(b)**, and pSpCas9-g11/13/14 **(c)**.

**(d)** Combined frequency distribution of indel sizes of three genes for the different constructs. The number of unique alleles (n) contributing to each category is indicated below the x-axis. Data points represent the frequency of individual alleles across callus lines. Error bars represent SEM.

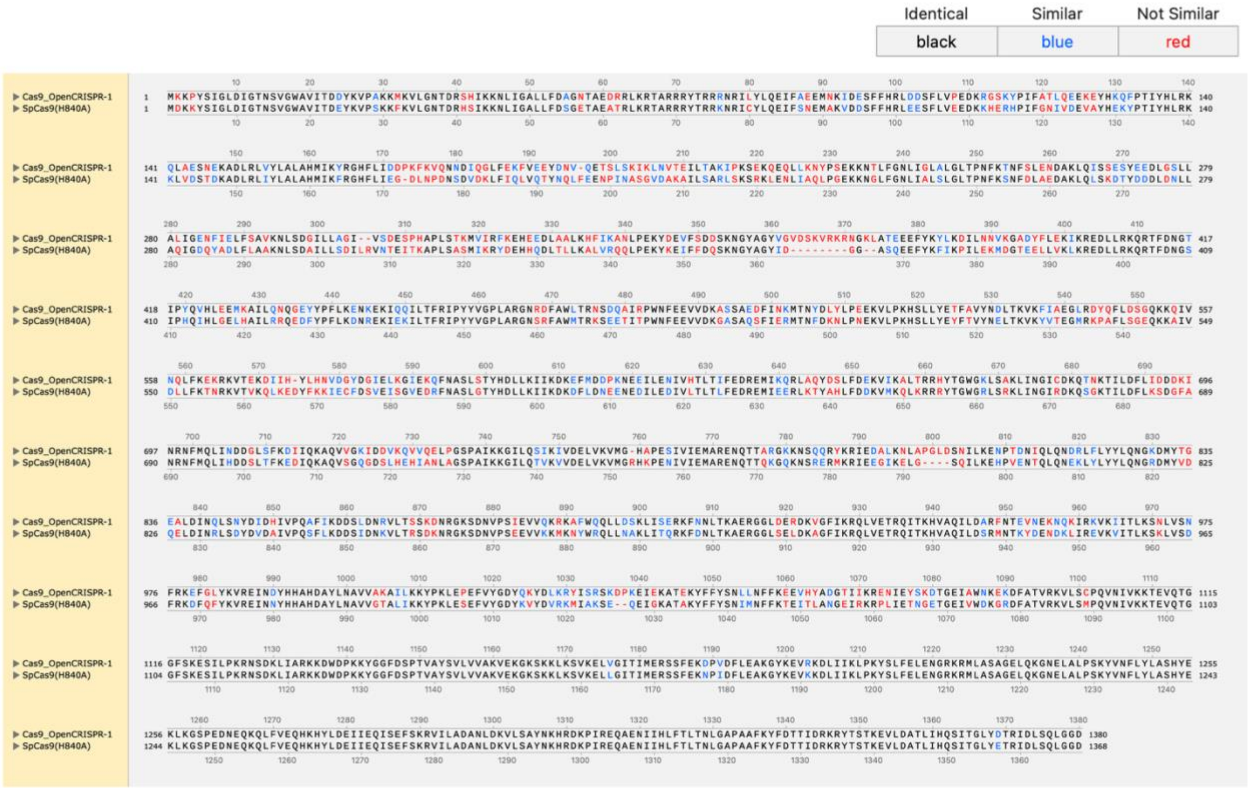


**Fig. S5: Alignment of OpCas9 and SpCas9(H840A) amino acid sequence.**

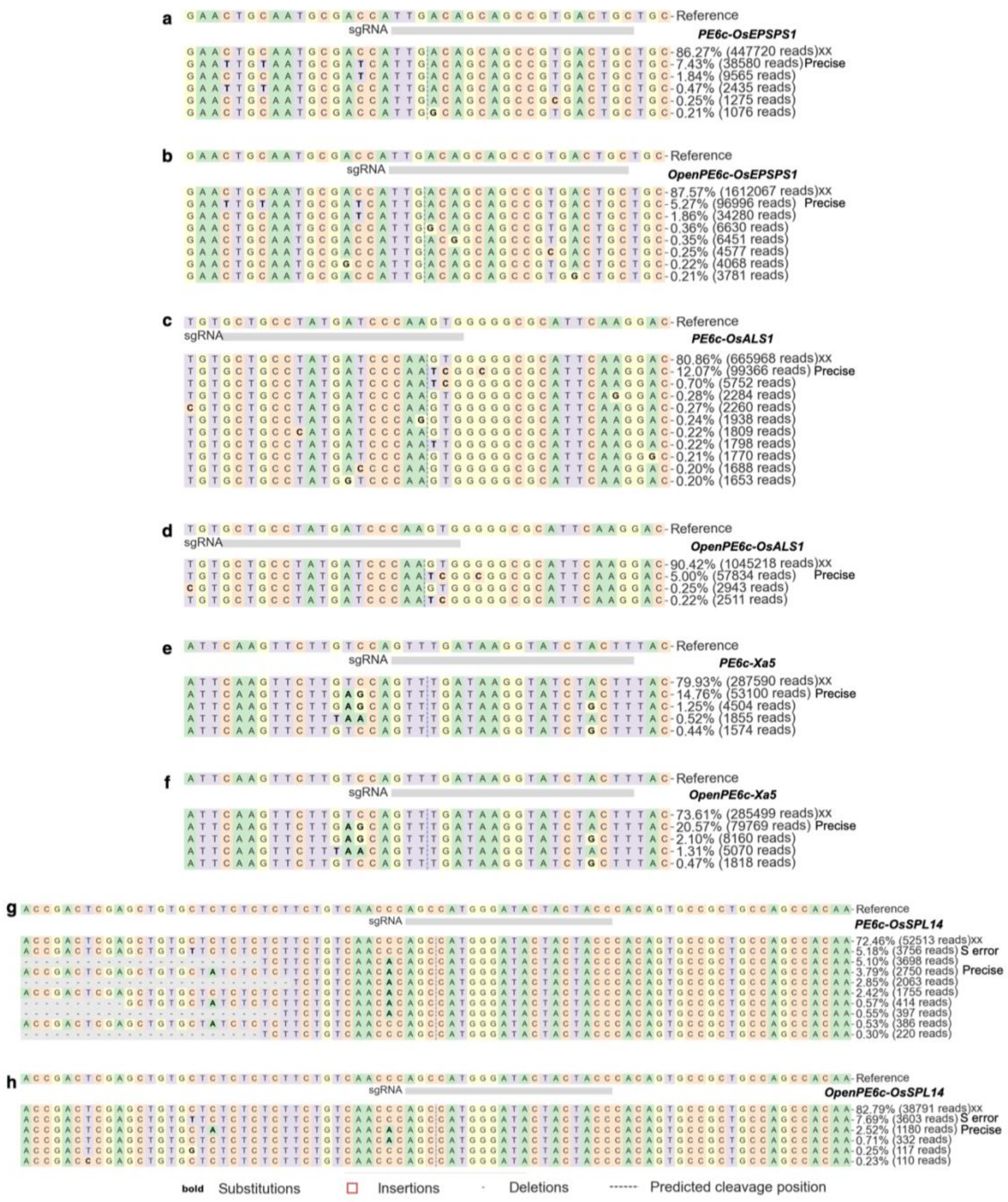

**Fig. S6: Deep amplicon sequencing analysis of PE outcomes.**
**(a–h)** CRISPResso2 visualization of the PE outcome for *OsEPSPS1* with PE6c **(a)** and OpenPE6c **(b)**, *OsALS1* with PE6c **(c)** and OpenPE6c **(d)**, *Xa5* with PE6c **(e)** and OpenPE6c **(f),** and *OsSPL14* with PE6c **(g)** and OpenPE6c **(h)**. The wild-type (WT) reference sequence is shown at the top with the sgRNA indicated by a gray bar. Substitutions are shown in bold, insertions are enclosed in red boxes, and deletions are represented by dashed lines. The vertical dashed line indicates the predicted cleavage position. Relative read percentages and counts are provided on the right; "xx" denotes the WT allele. Precisely edited reads and sequencing errors

(S errors) are labelled at the right side of the panel. Unlabeled reads are the imprecise edited alleles from protoplasts.

## Supplementary Tables

**Table S1: Genotypes of T0 plants from pOpCas9-g11.**

| **T0-pOpCas9-g11** | ***OsSWEET11a*** |
|---|---|
| 1 | B |
| 2 | B (+1/-4-bp) |
| 3 | B (-3/-5-bp) |
| 4 | B |
| 5 | B |
| 6 | B |
| 7 | M |
| 8 | B (-8/+1-bp) |
| 10 | B |
| 11 | B |
| 13 | M |
| 14 | B |
| 15 | M |
| 17 | B |
| 19 | B |
| 21 | B |
| 22 | B (+1-bp) |
| 23 | B |
| 24 | B |
| 25 | B |
| 26 | B |
| 28 | B (-3/+1-bp) |
| 29 | B |
| 30 | B (-2/+1-bp) |
| 31 | B (-3/+1-bp) |
| 32 | B |
| 33 | B |
| 35 | B (-3/-2-bp) |
| 36 | B |
| 37 | B |

B; Biallelic, M: Monoallelic, W: Wild type, Sequence is provided in brackets

**Table S2: Genotypes of T0 plants from pOpCas9-g14.**

| T0-pOpCas9-g14 | *OsSWEET14* |
|---|---|
| 1 | B(-4/-1-bp) |
| 2 | B(-2/+1-bp) |
| 3 | B(-8/-6-bp) |
| 4 | B(-4/-3/-1/+1-bp)* |
| 5 | B(-2/+1-bp) |
| 6 | B(-30/-9/-6/-3-bp)* |
| 7 | B(-2/-1/+1-bp)* |
| 8 | B(-3-bp) |
| 9 | B(-2/+1-bp) |
| 10 | B(-2/+1-bp) |
| 11 | B(-2/+1-bp) |
| 12 | B(-2/+1-bp) |
| 13 | B(-2/+1-bp) |
| 14 | B (-5/-3-bp) |
| 15 | B(-30/-6/-3/-1/+1-bp)* |
| 16 | B(-30/-6/-5/-3/+1-bp)* |
| 17 | B(-2/+1-bp) |
| 18 | B(-3-bp) |
| 19 | B(-5/-2-bp) |
| 20 | B(-1/+1-bp) |
| 21 | B(-1/+1-bp) |
| 22 | W |
| 23 | W |
| 24 | B(-5-bp) |
| 25 | B(-1-bp) |
| 26 | B(-3/-2/+1-bp)* |
| 27 | B(-4/-1/+1-bp)* |
| 28 | B(-3/-1-bp) |
| 29 | B(-2/+1-bp) |
| 30 | B(-4/-1/+1-bp)* |
| 31 | B(-1-bp) |

B; Biallelic, M: Monoallelic, W: Wild type, Sequence is provided in brackets
*These plants represent a mixture of T0 originated from single callus line.

**Table S3: Genotypes of T0 plants from pOpCas9-g11/13.**

| T0-pOpCas9-g11/13 | *OsSWEET11a* | *OsSWEET13* |
|---|---|---|
| 1 | B (-4/+1-bp) | B (-2/+1-bp) |
| 2 | B | W |
| 3 | B (-3/+1-bp) | B (-5/+1-bp) |
| 4 | B | B |
| 5 | M | M |
| 6 | B(+1-bp) | B (-3/+1-bp) |
| 9 | B | B |
| 10 | B | B |
| 11 | B | B |
| 12 | B | B |
| 13 | B | B |
| 14 | B | B |
| 15 | B | B |
| 16 | B | B |
| 19 | B | B |
| 20 | B | B |
| 21 | B (-4/+1-bp) | B +1-bp) |
| 22 | B | B |
| 23 | B | B |
| 24 | B | B |
| 25 | B | B |
| 26 | M | M |
| 27 | B | B |
| 29 | B | B |
| 30 | B | B |
| 31 | B | B |
| 32 | B | B |
| 33 | B | B |
| 34 | B | B |
| 35 | B | B |
| 36 | B | B |
| 37 | B | B |
| 38 | B | B |
| 39 | B | B |
| 40 | B | B |
| 41 | B | B |
| 42 | B | B |

| | | |
|---|---|---|
| 44 | B | B |
| 45 | B | B |
| 46 | W | W |
| 47 | B | B |
| 48 | B | B |
| 49 | M | B |
| 50 | B | B |
| 51 | B | B |
| 52 | B (-3/-2-bp) | B (+1-bp) |
| 53 | B | B |

B; Biallelic, M: Monoallelic, W: Wild type, Sequence is provided in brackets

**Table S4: Genotypes of T0 plants from pOpCas9-g11/13/14.**

| T0-pOpCas9-g11/13/14 | *OsSWEET11a* | *OsSWEET13* | *OsSWEET14* |
|---|---|---|---|
| 1 | M (W/-2/+1-bp) | M (W/+1-bp) | M (W/-1/+1-bp) |
| 2 | B (+1-bp) | B (-3/+1-bp) | B (-3/-1-bp) |
| 3 | B (-2/-5-bp) | B (-4/+1-bp) | B (-48/+1-bp) |
| 4 | B (-7/+1-bp) | B (-3/+1-bp) | B (-2/-1-bp) |
| 5 | B (-7/+3-bp) | W (W) | B (-2-bp) |
| 6 | B (-4/+1-bp) | B (-3/+1-bp) | B (-1/+1-bp) |
| 7 | B (+1-bp) | B (-5/-2/+1-bp) | B (-2/+1-bp) |
| 8 | B (-4/-3/+1-bp) | B (+1-bp) | B (-3/-2/+1-bp) |
| 9 | M (W/-2/+1-bp) | B (-4/+1-bp) | B (-3/-2/-1-bp) |
| 10 | B (-9/-8/-4/-3/+1-bp) | B (+1-bp) | B (-3/-2/-1/+1-bp) |
| 11 | B (-3/+1-bp) | M (W/+1-bp) | B (-2/-1/+1-bp) |
| 12 | B (-11/+1-bp) | B (-14/+1-bp) | B (-1-bp) |
| 13 | B (-4/+1-bp) | B (-3/+1-bp) | B (-1/+1-bp) |
| 14 | B (-9/-3/-2/+1-bp) | M (W/-2/+1-bp) | B (-4/-2/+1-bp) |
| 15 | B (-7/-3/+1-bp) | B (-3/+1-bp) | B (-3/-1-bp) |
| 16 | M (W/-16/-5/+1-bp) | B (-5/+1-bp) | B (-4/-3/-1-bp) |
| 17 | B | B | B (-4/-3-bp) |
| 18 | B | B | M (W/-5/-1-bp) |
| 19 | B | B | B (-2/-1-bp) |
| 20 | B | B | B (-2/-1-bp) |
| 21 | B | B | B (-1-bp) |
| 22 | B (-8/-3-bp) | B (+1/-2-bp) | B (-5/-1-bp) |
| 23 | B | B | M (W/-3/-2-bp) |
| 24 | M | B | B (-3/-2-bp) |
| 25 | M | W | B (-2/-1-bp) |
| 26 | B | B | B (-6/-5/-1-bp) |
| 27 | B | B | B (-2/+1-bp) |
| 28 | B | B | B (-3/-2-bp) |
| 29 | B | B | B (-2-bp) |
| 30 | W | W | B (-3/+1-bp) |
| 31 | M | B | B (-3/-2-bp) |
| 32 | M | B | B (-3/-1/+1-bp) |
| 33 | M | B | B (-3/-1-bp) |
| 34 | M | B | B (-12/+1-bp) |
| 35 | M | B | B (-4/-3/-1-bp) |
| 36 | W | W | W (W) |
| 37 | B | B | B (-3/-1-bp) |

| | | | |
|---|---|---|---|
| 38 | B | B | B (-3/-1-bp) |
| 39 | B | B | B (-5/-1-bp) |
| 40 | B | B | B (-2/+1-bp) |
| 41 | M | B | B (-2-bp) |
| 42 | M | B | B (-3/-2-bp) |
| 43 | B | B | B (-2/-1-bp) |

B; Biallelic, M: Monoallelic, W: Wild type, Sequence is provided in brackets
*These plants represent a mixture of T0 originated from single callus line.

**Table S5: Genotypes of T0 plants from pSpCas9-g11/13/14.**

| **T0-pSpCas9-g11-13-14** | ***OsSWEET11a*** | ***OsSWEET13*** | ***OsSWEET14*** |
|---|---|---|---|
| 2 | B (-3/+1-bp) | B (+1-bp) | B (-2/-4-bp) |
| 8 | M (W/-4/-3/+1-bp)* | B (+1-bp) | B (-1/+1-bp) |
| 9 | B (-14/-3-bp) | B (-5/+1-bp) | B (-2/+1-bp) |
| 10 | B (+1-bp) | B (-4/+1-bp) | B (-1-bp) |
| 11 | B (-1/+1-bp) | M (W/+1-bp) | B (-2/-1-bp) |
| 14 | B (-3/+1-bp) | B (+1-bp) | B (-2/-4-bp) |
| 15 | B (-2/+1-bp) | B (-4/+1-bp) | B (-2/-1-bp) |

B; Biallelic, M: Monoallelic, W: Wild type, Sequence is provided in brackets
*These plants represent a mixture of T0 originated from single callus line.

**Table S6: Genotypes of T0 plants from pOpCas9-gOpSWT11.**

| pOpCas9-gOpSWT11 | *OsSWEET11a* |
|---|---|
| 1 | B (-6/-3-bp) |
| 2 | B (-14/-3-bp) |
| 3 | M (W/-4/+1-bp)* |
| 4 | B (-3/-1-bp) |
| 5 | B (-9/-6/-3/+1-bp)* |
| 6 | B (-2/+1-bp) |
| 7 | B (-2/+1-bp) |
| 8 | B (-4/-3/+1-bp)* |
| 9 | B (+1-bp) |
| 10 | M (W/-2/+1-bp)* |
| 11 | B (-6/-3/-2/+1-bp)* |
| 12 | M (W/-2/+1-bp)* |
| 13 | B (-19/+1-bp) |
| 14 | W |
| 15 | B (-7/+1-bp) |
| 16 | B (-1/+1-bp) |
| 17 | B (-23/-5/-2-bp)* |
| 18 | B (-3/+1-bp) |
| 19 | B (-3/+1-bp) |
| 20 | B (-12/-10/-6/-5/-3-bp)* |
| 21 | B (-5/-3/-2/+1-bp)* |
| 22 | B (-6/-4/-3/+1-bp)* |
| 23 | B (-3/-1-bp) |
| 24 | B (-1/+1-bp) |
| 25 | B (-4/-3/+1-bp) |
| 26 | B (-5/-4/-3/-2/+1-bp)* |
| 27 | B (-11/-3-bp) |
| 28 | B (-2-bp) |
| 29 | B (-1/+1-bp) |
| 30 | B (-6/-5/-2/+1-bp)* |

B; Biallelic, M: Monoallelic, W: Wild type, Sequence is provided in brackets
*These plants represent a mixture of T0 originated from single callus line.

**Table S7: Genotypes of T0 plants from pOpCas9-gOpSWT13.**

| pOpCas9-gOpSWT13 | *OsSWEET13* |
|---|---|
| 1 | B (+1-bp) |
| 2 | B (+1-bp) |
| 3 | B (+1-bp) |
| 4 | B (+1-bp) |
| 5 | B (-5/-4/-2-bp)* |
| 6 | B (-13/+1-bp) |
| 7 | M (W/+1-bp) |
| 8 | B (+1-bp) |
| 9 | B (+1-bp) |
| 10 | B (-4/+1-bp) |
| 11 | B (-2/+1-bp) |
| 12 | B (+1-bp) |
| 13 | B (+1-bp) |
| 14 | B (+1-bp) |
| 15 | B (-3/+1-bp) |
| 16 | M (W/+1-bp) |
| 17 | B (+1-bp) |
| 18 | B (-3/+1-bp) |
| 19 | B (-3/+1-bp) |
| 20 | B (+1-bp) |
| 21 | B (+1-bp) |
| 22 | B (-4/+1-bp) |
| 23 | B (+1-bp) |
| 24 | B (-53/+1-bp) |
| 25 | W |
| 26 | B (+1-bp) |
| 27 | W |
| 28 | B (-5/+1-bp) |
| 29 | W |
| 30 | B (-13/+1-bp) |

B; Biallelic, M: Monoallelic, W: Wild type, Sequence is provided in brackets
*These plants represent a mixture of T0 originated from single callus line.

**Table S8: Genotypes of T0 plants from pOpCas9-gOpSWT14.**

| **pOpCas9-gOpSWT14** | ***OsSWEET14*** |
|---|---|
| 1 | B (-3/-2-bp) |
| 2 | B (-3/-2-bp) |
| 3 | B (-3/-2-bp) |
| 4 | B (-2/+1-bp) |
| 5 | B (-3-bp) |
| 6 | B (-1-bp) |
| 7 | B (-2/-1/+1-bp)* |
| 8 | B (-2/-5-bp) |
| 9 | B (-2/-1-bp) |
| 10 | B (-4-bp) |
| 11 | B (-3/-2/+1-bp)* |
| 12 | B (-2/-1-bp) |
| 13 | B (-3/-4-bp) |
| 14 | B (-2/-1-bp) |
| 15 | B (-5/-3/-2/-1/+1-bp)* |
| 16 | B (-5-bp) |
| 17 | B (-12/-4/-1/+1-bp)* |
| 18 | B (-1/+1-bp) |
| 19 | B (-3/-2-bp) |
| 20 | B (-2/-1-bp) |
| 21 | B (-2/-1-bp) |
| 22 | M (W/-3/-2/-1-bp)* |
| 23 | B (-3/+1-bp) |
| 24 | B (-1-bp) |
| 25 | B (-2/-1-bp) |
| 26 | W |
| 27 | B (-2/-1-bp) |
| 28 | B (-48/-3/+1-bp)* |
| 29 | B (-13/-3-bp) |
| 30 | M (W/+1-bp) |

B; Biallelic, M: Monoallelic, W: Wild type, Sequence is provided in brackets
*These plants represent a mixture of T0 originated from single callus line.

**Table S9: Oligos used in this study**

| Name | Sequence (5´ to 3´) | Purpose |
|---|---|---|
| gSwt11a-F6<br>gSwt11a-R6 | tgttGGTGTACAAGAAGAAGTCGA<br>aaacTCGACTTCTTCTTGTACACC | Oligo for OsSWEET11a sgRNA cloning |
| gSwt13-F6<br>gSwt13-R6 | gtgtGATCTACAAGAGCAAGTCGA<br>aaacTCGACTTGCTCTTGTAGATC | Oligo for OsSWEET13 sgRNA cloning |
| gSwt14-F6<br>gSwt14-R6 | tgttGTTCCAGTCGGTACCCTACG<br>aaacCGTAGGGTACCGACTGGAAC | Oligo for OsSWEET14 sgRNA cloning |
| MisSWT11-F1<br>MisSWT11-R1 | CTCTTTCCCTACACGACgctcttccgatctTCTCTGACGACGAACTGGAA<br>ctggagttcagacgtgtgctcttccgatctGTAGAGGACGATGTAGGCGG | For genotyping and deep-amplicon sequencing of OsSWEET11a |
| MisSWT13-F1<br>MisSWT13-R1 | CTCTTTCCCTACACGACgctcttccgatctACCTCATATCCTTCACGACCT<br>ctggagttcagacgtgtgctcttccgatctATCAGCGCGTAGAAGATCCA | For genotyping and deep-amplicon sequencing of OsSWEET13 |
| MisSWT14-F1<br>MisSWT14-R1 | CTCTTTCCCTACACGACgctcttccgatctAAAACACTCCCCGTCTCTGT<br>ctggagttcagacgtgtgctcttccgatctTGGTGATGAGGAGGCACTCG | For genotyping and deep-amplicon sequencing of OsSWEET14 |
| pegXa5-F1<br>pegXa5-R1<br>extXa5-F1<br>extXa5-R1<br>ngXa5-F1<br>ngXa5-R1 | tgcaAAGTAGATACCTTATCAAAC<br>aaacGTTTGATAAGGTATCTACTT<br>gtgcGCCATTCAAGTTCTTGagCAGTTTGATAAGGTATCAGGAATAA<br>cgcgTTATTCCTGATACCTTATCAAACTGctCAAGAACTTGAATGGC<br>gataAGTTCTTGagCAGTTTGATA<br>aaacTATCAAACTGctCAAGAACT | To make pegRNA/ngRNA in entry vectors to target *TFIIAγ5* |
| pegOsEPSPS-F1<br>pegOsEPSPS-R1<br>extOsEPSPS-F1<br>extOsEPSPS-R1<br>ngOsEPSPS-F1<br>ngOsEPSPS-R1 | tgcaGCAGTCACGGCTGCTGTCAA<br>aaacTTGACAGCAGCCGTGACTGC<br>gtgcTGGAAtTGtAATGCGAtCATTGACAGCAGCCGTGAAGAAATAT<br>cgcgATATTTCTTCACGGCTGCTGTCAATGaTCGCATTaCAaTTCCA<br>gataTGTTGAGAAGGATGCGAAAG<br>aaacCTTTCGCATCCTTCTCAACA | To make pegRNA/ngRNA in entry vectors to target *OsEPSPS1* |

| | | |
|---|---|---|
| pegOsALS-F1<br>pegOsALS-R1<br>extOsALS-F1<br>extOsALS-R1<br>ngOsALS-F1<br>ngOsALS-R1 | tgcaGCTGCCTATGATCCCAAGTG<br>aaacCACTTGGGATCATAGGCAGC<br>gtgcGCCgCCgaTTGGGATCATAGGCTTAAACAG<br>cgcgCTGTTTAAGCCTATGATCCCAAtcGGcGGC<br>gataTCCTTGAATGCGCCgCCgaT<br>aaacAtcGGcGGCGCATTCAAGGA | To make pegRNA/ngRNA in entry vectors to target *OsALS1* |
| pegSPL14-F1<br>pegSPL14-R1<br>extSPL14-F1<br>extSPL14-R1<br>ngSPL14-F1<br>ngSPL14-R1 | tgcaGGTAGTAGTATCCCATGGCT<br>aaacAGCCATGGGATACTACTACC<br>gtgcTGTGCTaTCTCTCTTCTGTCAACaCAGCCATGGGATACTAAGGAATAA<br>cgcgTTATTCCTTAGTATCCCATGGCTGtGTTGACAGAAGAGAGAtAGCACA<br>gataGCTGGCCCAAATCTCCCTCC<br>aaacGGAGGGAGATTTGGGCCAGC | To make pegRNA/ngRNA in entry vectors to target *OsSPL14* |
| Xa5-F1<br>Xa5-R2 | CTCTTTCCCTACACGACgctcttccgatctCATTGGCATGTGCCTCACTG<br>ctggagttcagacgtgtgctcttccgatctCTCCAAGGCTTCCGTCATAG | Deep amplicon sequencing of *xa5* PE edits |
| OsEPSPS1-F3<br>OsEPSPS1-R3 | ctggagttcagacgtgtgctcttccgatctGGTGGCAAGTTTCCTGTT<br>CTCTTTCCCTACACGACgctcttccgatcCCCCATGAATTCCATACAT | Deep amplicon sequencing of *EPSPS1* PE edits |
| OsALS-F5<br>OsALS-R5 | CTCTTTCCCTACACGACgctcttccgatctGCCGCCATCAAGAAGA<br>ctggagttcagacgtgtgctcttccgatctAGTCCTGCCATCACCAT | Deep amplicon sequencing of *ALS1* PE edits |
| OsSPL14-F2<br>OsSPL14-R2 | CTCTTTCCCTACACGACgctcttccgatctAAGGTAGCTCTTCTTCAGGG<br>ctggagttcagacgtgtgctcttccgatctGGGGCTTGGTGCCATGTAGC | Deep amplicon sequencing of SPL14 PE edits |
| OpenCas9-F2<br>OpCas9-H850A-R1 | ACAGGATGGGGCAAGCTCAGCG<br>GCtTGAGGGACGATcgcATCGATGTCATAG | To screen for the presence of transgene on OC-1 lines |
| Truseq-R49<br>Truseq-R50<br>Truseq-R51<br>Truseq-R52 | CAAGCAGAAGACGGCATACGAGAT**<u>CGAGTAAT</u>**GTGACTGGAGTTCAGACGTGTGCT<br>CAAGCAGAAGACGGCATACGAGAT**<u>TCTCCGGA</u>**GTGACTGGAGTTCAGACGTGTGCT<br>CAAGCAGAAGACGGCATACGAGAT**<u>AATGAGCG</u>**GTGACTGGAGTTCAGACGTGTGCT<br>CAAGCAGAAGACGGCATACGAGAT**<u>GGAATCTC</u>**GTGACTGGAGTTCAGACGTGTGCT | dual barcoded oligos for deep sequencing library preperation. Barcodes are bold and underlined |

| | | |
|---|---|---|
| Truseq-R53 | CAAGCAGAAGACGGCATACGAGAT**TTCTGAAT**GTGACTGGAGTTCAGACGTGTGCT | |
| Truseq-R54 | CAAGCAGAAGACGGCATACGAGAT**ACGAATTC**GTGACTGGAGTTCAGACGTGTGCT | |
| Truseq-R55 | CAAGCAGAAGACGGCATACGAGAT**AGCTTCAG**GTGACTGGAGTTCAGACGTGTGCT | |
| Truseq-R56 | CAAGCAGAAGACGGCATACGAGAT**GCGCATTA**GTGACTGGAGTTCAGACGTGTGCT | |
| Truseq-R57 | CAAGCAGAAGACGGCATACGAGAT**CATAGCCG**GTGACTGGAGTTCAGACGTGTGCT | |
| Truseq-R58 | CAAGCAGAAGACGGCATACGAGAT**TTCGCGGA**GTGACTGGAGTTCAGACGTGTGCT | |
| Truseq-R59 | CAAGCAGAAGACGGCATACGAGAT**GCGCGAGA**GTGACTGGAGTTCAGACGTGTGCT | |
| Truseq-R60 | CAAGCAGAAGACGGCATACGAGAT**CTATCGCT**GTGACTGGAGTTCAGACGTGTGCT | |
| TruSeq-F1 | AATGATACGGCGACCACCGAGATCTACAC**TATAGCCT**ACACTCTTTCCCTACACGACGCTCT | |
| TruSeq-F2 | AATGATACGGCGACCACCGAGATCTACAC**ATAGAGGC**ACACTCTTTCCCTACACGACGCTCT | |
| TruSeq-F3 | AATGATACGGCGACCACCGAGATCTACAC**CCTATCCT**ACACTCTTTCCCTACACGACGCTCT | |
| TruSeq-F4 | AATGATACGGCGACCACCGAGATCTACAC**GGCTCTGA**ACACTCTTTCCCTACACGACGCTCT | |
| TruSeq-F5 | AATGATACGGCGACCACCGAGATCTACAC**AGGCGAAG**ACACTCTTTCCCTACACGACGCTCT | |
| TruSeq-F6 | AATGATACGGCGACCACCGAGATCTACAC**TAATCTTA**ACACTCTTTCCCTACACGACGCTCT | |
| TruSeq-F7 | AATGATACGGCGACCACCGAGATCTACAC**CAGGACGT**ACACTCTTTCCCTACACGACGCTCT | |
| TruSeq-F8 | AATGATACGGCGACCACCGAGATCTACAC**GTACTGAC**ACACTCTTTCCCTACACGACGCTCT | |

## Supplementary Sequences

Supplementary sequence S1

NLS-NLS-monocot-OpenCas9-NLS-NLS

atgaagaggacagccgatggcagcgagttcgagagccctaagaaaaagaggaaggtctcaggcg
gctcatctggcgggtcaaagcgcacagccgacggctctgagttcgagagcAGGccgaaaaagaa
gcgcaaagtgtcaggcggctcttcaggcggcagcATGAAGAAACCTTATTCTATcGGGCTGGAT
ATTGGAACAAATTCCGTGGGTTGGGCCGTGATTACAGATGACTACAAGGTcCCGGCGAAGAAGA
TGAAGGTTCTTGGCAACACCGACCGCAGCCATATCAAGAAAAATTTGATTGGGGCTTTGCTGTT
TGATGCTGGCAATACAGCAGAGGACAGGCGcTTgAAGAGGACCGCcAGACGGCGCTACACCAGA
AGGCGCAACCGcATTCTGTACTTGCAAGAGATTTTCGCCGAGGAGATGAACAAGATcGACGAAT
CATTCTTCCACCGCTTGGACGACTCCTTTTTGGTGCCTGAGGACAAGCGGGGATCAAAGTATCC
AATTTTTGCAACACTGCAGGAGGAGAAGGAGTACCACAAGCAGTTCCCGACGATcTATCATTTG
CGGAAGCAACTcGCAGAATCCAATGAGAAGGCTGACCTTCGCCTCGTTTATTTgGCGCTCGCTC
ACATGATTAAGTACAGGGGCCACTTCCTGATCGACGACCCAAAGTTCAAGGTTCAGAACAATGA
TATcCAAGGGCTTTTTGAGAAGTTTGTCGAGGAGTATGATAACGTGCAGGAGACTAGCCTcTCC
AAGATTAAGCTGAATGTTACTGAAATTTTGACCGCGAAGATTCCTAAATCAGAGAAACAAGAGC
AGCTCCTCAAGAATTACCCGAGCGAGAAGAAGAACACGCTGTTTGGAAATCTGATTGGCCTTGC
GCTGGGCCTGACACCAAATTTCAAGACAAACTTCTCACTcGAGAATGACGCTAAACTCCAGATC
AGcTCTGAATCTTATGAAGAAGATTTgGGCTCTCTGCTGGCGCTCATTGGTGAAAATTTTATTG
AACTcTTTTCTGCTGTCAAAAACCTCTCAGATGGcATTCTTCTTGCgGGAATTGTgAGcGACGA
GAGCCCTCACGCGCCACTCTCTACCAAGATGGTCATTCGcTTCAAGGAGCATGAAGAAGATCTC
GCTGCACTCAAGCATTTTATCAAAGCGAATCTCCCAGAGAAGTATGATGAGGTCTTCTCCGACG
ATTCTAAAAACGGATATGCAGGATACGTTGGCGTCGACTCCAAAGTgCGcAAGAGAAATGGAAA
ACTGGCGACGGAGGAGGAgTTCTACAAATACCTTAAGGACATcCTCAACAATGTTAAAGGCGCC
GACTATTTCcTcGAGAAGATcAAGCGcGAGGACCTCCTCAGAAAACAACGCACCTTCGACAACG
GCACCATCCCCTATCAGGTTCATCTGGAAGAGATGAAAGCAATTTTGCAGAATCAGGGGGAGTA
CTACCCTTTCTTGAAGGAGAATAAGGAAAAAATCCAACAGATcTTGACGTTTCGCATcCCATAC
TATGTCGGACCACTTGCCCGCGGCAACAGAGATTTTGCTTGGCTGACAAGAAACAGCGACCAGG
CAATCCGGCCGTGGAATTTCGAGGAAGTTGTGGACAAGGCATCTTCGGCTGAAGACTTCATTAA
TAAAATGACTAACTACGATCTGTACCTCCCCGAGGAAAAGGTCCTGCCGAAACACTCCCTCCTC
TATGAGACATTCGCTGTGTATAACGAcCTcACCAAaGTGAAGTTTATtGCGGAGGGTCTTCGcG
ATTACCAATTTCTcGACAGcGGGCAGAAGAAGCAAATTGTTAATCAGCTCTTTAAAGAGAAACG
GAAAGTGACTGAAAAAGATATcATCCACTATCTGCACAACGTCGACGGCTATGATGGTATcGAG
CTgAAGGGCATTGAGAAACAGTTCAATGCCAGcCTCTCTACATACCATGATcTcTTGAAAATTA
TCAAAGATAAGGAGTTTATGGACGATCCTAAGAACGAAGAAATCCTGGAGAACATCGTGCACAC
CcTcACAATCTTTGAGGATAGGGAGATGATCAAGCAGAGGTTGGCACAATATGACTCTCTGTTT
GACGAGAAGGTCATCAAGGCGCTCACGCGCCGcCATTATACAGGATGGGGCAAGCTCAGCGCAA
AGTTGATTAACGGGATcTGcGATAAACAGACAAACAAGACCATCTTGGATTTCcTcATCGACGA
TGACAAGATCAACAGGAACTTCATGCAGCTcATCAACGATGATGGcCTcTCATTTAAGGATATc
ATACAAAAGGCCCAGGTGGTCGGCAAGATCGATGATGTGAAGCAGGTCGTGCAAGAACTCCCTG
GTAGCCCTGCTATcAAGAAGGGTATTCTGCAAAGCATCAAGATCGTCGATGAATTGGTCAAAGT
cATGGGCCATGCTCCTGAAAGCATCGTTATTGAAATGGCGAGGGAGAACCAAACAACAGCCCGC
GGGAAGAAAAATAGCCAACAGCGGTACAAGAGGATCGAAGACGCTCTGAAAAATCTGGCCCCCG
GcCTCGACTCAAATATcCTTAAGGAAAATCCTACTGACAATATCCAGCTcCAGAACGACAGACT
TTTCTTTACTACCTTCAAAATGGGAAAGACATGTACACGGGAGAGGCCCTCGATATcAATCAA

TTGTCCAACTATGACATCGATCACATCGTCCCTCAaGCCTTTATTAAGGACGATAGCCTCGATA
ATAGGGTcCTCACTTCTTCGAAGGATAATCGGGGCAAATCCGACAACGTGCCGAGCATCGAGGT
GGTTCAGAAGAGGAAGGCATTCTGGCAGCAACTGCTcGATTCTAAACTTATcTCGGAAAGGAAG
TTCAACAACCTcACGAAGGCCGAGAGAGGTGGCCTCGATGAAAGGGACAAAGTTGGTTTCATCA
AGCGCCAGCTGGTGGAAACGAGACAAATTACTAAGCATGTTGCGCAGATcCTGGATGCAAGATT
CAATACTGAAGTGAATGAAAAGAATCAGAAAATTAGGAAGGTCAAGATcATCACAcTcAAATCA
AACcTcGTgTCGAACTTTCGGAAGGAATTTGGTTTGTACAAGGTCCGGGAGATcAATGATTACC
ACCACGCCCATGACGCCTACcTcAACGCAGTGGTCGCCAAGGCCATCcTcAAGAAGTACCCCAA
GCTcGAGCCAGAGTTCGTGTATGGAGATTATCAGAAATACGATCTTAAGCGcTACATTAGcCGC
TCTAAAGATCCAAAGGAGATTGAGAAGGCTACCGAGAAATATTTCTTCTATTCCAACCTGCTcA
ATTTCTTCAAaGAGGAAGTGCATTACGCCGATGGGACTATTATTAAACGGGAGAACATCGAATA
TTCAAAGGATACTGGTGAGATCGCCTGGAACAAAGAAAAGGACTTCGCGACCGTGcGcAAAGTC
CTCTCCTGCCCGCAAGTTAACATCGTTAAGAAAACAGAAGTGCAAACTGGCGGCTTCTCGAAAG
AGTCTATCCTTCCGAAGCGcAACAGcGATAAGTTGATCGCACGCAAAAAGGACTGGGACCCAAA
GAAATATGGTGGATTCGACTCACCGACTGTTGCGTACAGCGTCCTCGTCGTGGCAAAGGTGGAA
AAGGGcAAGTCTAAAAAGCTcAAGTCCGTTAAAGAACTCGTTGGCATcACCATCATGGAGAGGT
CGTCGTTCGAGAAGGACCCCGTGGATTTCCTGGAAGCCAAGGGCTACAAGGAaGTTAGAAAGGA
CCTCATCATCAAGCTCCCCAAGTATAGCCTGTTCGAGCTGGAAAACGGACGCAAGAGGATGCTG
GCGTCGGCCGGCGAGTTGCAAAAGGGAAACGAGCTgGCCCTGCCAAGCAAATATGTCAACTTCC
TCTACCTTGCTTCACACTACGAGAAGCTGAAGGGGTCGCCGGAAGATAATGAGCAAAAACAACT
CTTCGTGGAGCAGCATAAGCACTACcTcGATGAGATCATCGAGCAGATCAGCGAATTTTCAAAG
AGAGTGATTcTcGCTGATGCTAATCTcGATAAGGTGCTCTCCGCCTACAATAAACATCGGGACA
AGCCCATTAGAGAACAAGCTGAGAATATTATCCACCTCTTCACCCTTACCAATTTGGGTGCGCC
CGCGGCGTTCAAGTACTTTGACACGACAATTGATCGcAAAcGcTACACTTCAACGAAAGAAGTg
CTTGACGCCACGCTCATCCATCAATCCATCACTGGTCTTTATGAcACGCGcATTGATCTGTCTC
AacTcGGTGGCGACGAGGGGGCCGACAAACGcACCGCGGACGGGAGCGAATTTGAATCTgggag
cggtcctgctgcaaagagggtcaaacttgattcttcaggcggcagcGAGGAACAGGCCCGGAAG
GCGAAAGTGAACAATGAGAAGAAAACAGAAATCGTTAAACCTGAGAGCTGCTCAAACGAGGGAG
AgGTTAAGGATCTTAAGAGGAAAGACTCTGAGGATGGCAACGAAGGAGAGGAAGAGGAAGCTAG
CTCAAAACCTAAGAAACCAAAGGTGGCACTCTCACATCTCCAAGACATTGACGACACCGAAGCA
GACCAAGAAGAAGAAtga

Supplementary sequence S2

Open gRNA scaffold
GTTTTAGAGCTGTGTTGAAAAACACAGCAAGTTAAAATAAGGCTTTGTCCGTATCCAACTTGAA
AAAGTGAGCACCGATTCGGTGC
